# Supplementary material for: Development of a Novel Endometrial Signature Based on Endometrial microRNA for Determining the Optimal Timing for Embryo Transfer
Source: Biomedicines. 2024 Mar 21;12(3):700. doi: 10.3390/biomedicines12030700 (PMC10968378; doi:10.3390/biomedicines12030700)
Supplement: Supplementary file 1 [file biomedicines-12-00700-s001.zip › Figure S1.pdf]

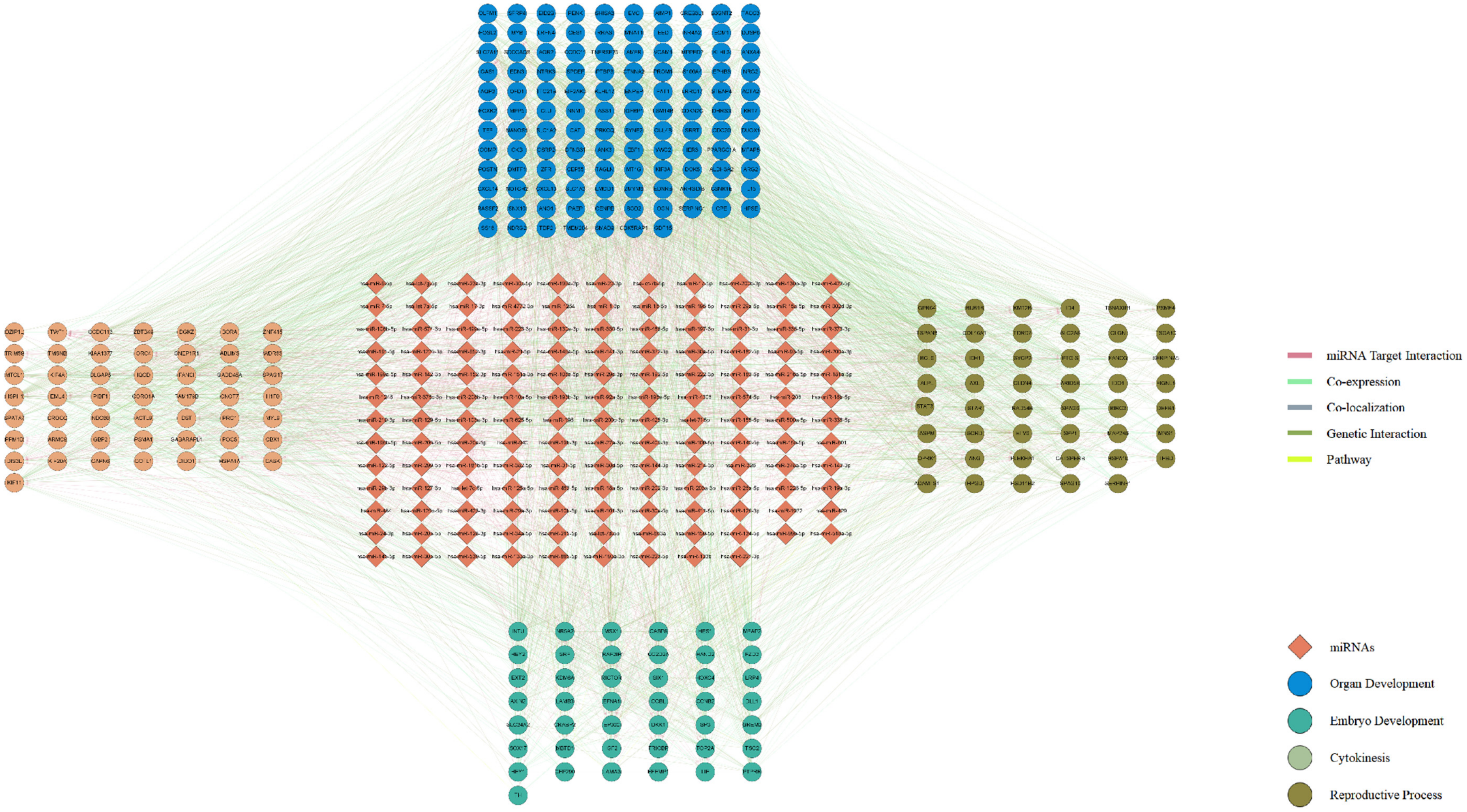

Figure S1. miRNA-gene interaction network. The data expressed in tabular form is shown in Supplemental Table S2.
