# Supplementary material for: Development of a Novel Endometrial Signature Based on Endometrial microRNA for Determining the Optimal Timing for Embryo Transfer
Source: Biomedicines. 2024 Mar 21;12(3):700. doi: 10.3390/biomedicines12030700 (PMC10968378; doi:10.3390/biomedicines12030700)
Supplement: Supplementary file 1 [file biomedicines-12-00700-s001.zip › Figure S2.pdf]

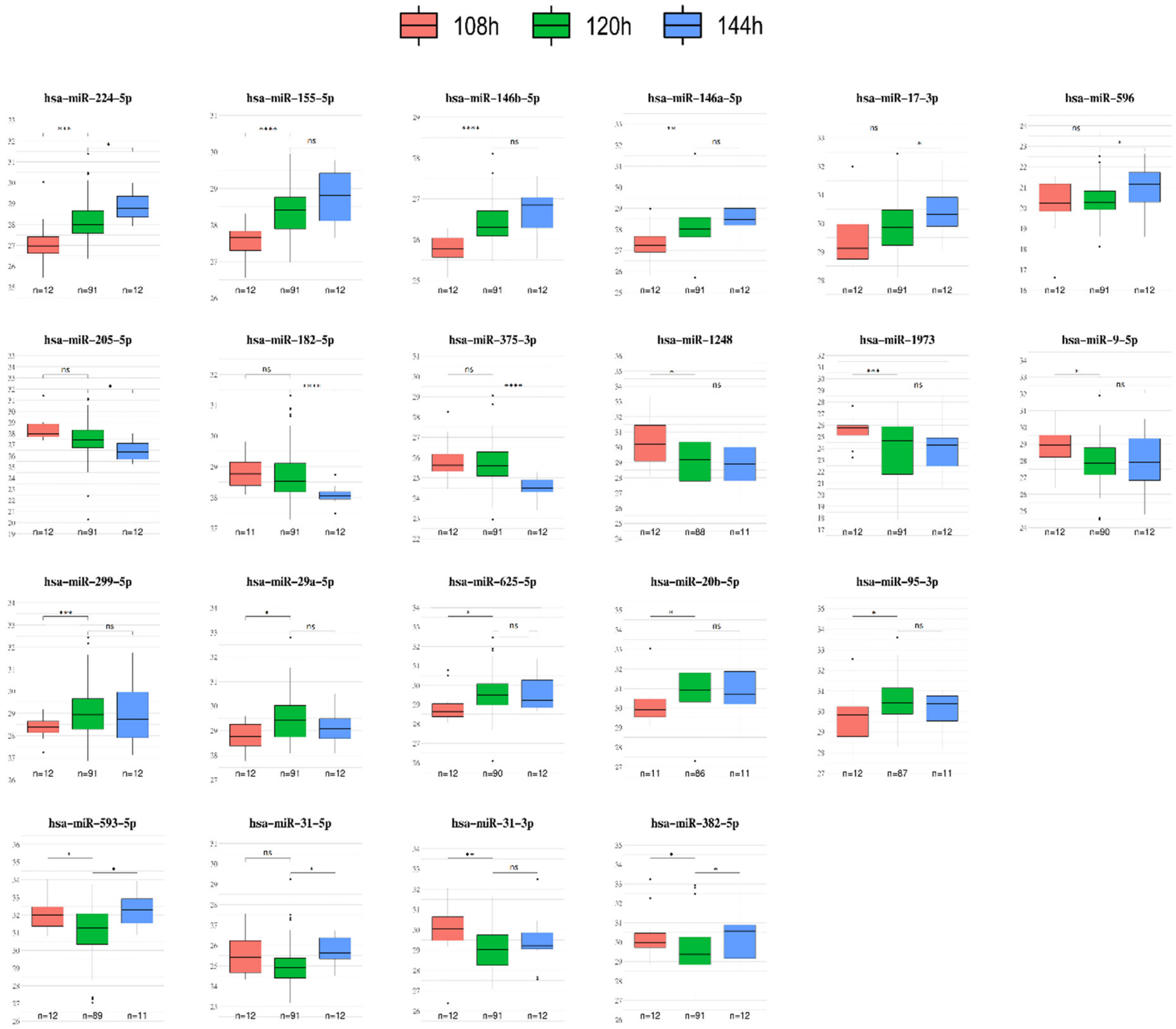

Figure S2. The differentially expressed miRNAs. miRNA signatures either for 120±5hrs vs. 108±5hrs or for 120±5hrs vs. 144±5hrs. The differentially expressed miRNAs were selected by the log2(fold-change) ≥ ±0.585 with p < 0.05. Y-axis denotes Cq, and X-axis represents the number of cases. Asterisks in statistics: ns denotes $p > 0.05$, * denotes $p \leq 0.05$, ** denotes $p \leq 0.01$, *** denotes $p \leq 0.001$, and **** denotes $p \leq 0.0001$.
