# Supplementary material for: Development of a Novel Endometrial Signature Based on Endometrial microRNA for Determining the Optimal Timing for Embryo Transfer
Source: Biomedicines. 2024 Mar 21;12(3):700. doi: 10.3390/biomedicines12030700 (PMC10968378; doi:10.3390/biomedicines12030700)
Supplement: Supplementary file 1 [file biomedicines-12-00700-s001.zip › Table S1.pdf]

Supplemental Table S1. Multiple group comparison for characteristics of patients at the time of biopsy.

Multiple group comparison (Training & Testing set, N = 150)

Age	Post hoc test (p-value) Bonferroni	Multiple group test (p-value) Kruskal Wallis
**108±5hrs vs 120±5hrs**	0.6505	
**108±5hrs vs 144±5hrs**	0.0072	0.0107
**120±5hrs vs 144±5hrs**	0.0204	

No. of previous implantation failure	Post hoc test (p-value) Bonferroni	Multiple group test Kruskal Wallis
**108±5hrs vs 120±5hrs**	0.0085	
**108±5hrs vs 144±5hrs**	0.0313	0.0361
**120±5hrs vs 144±5hrs**	1	

P4 Level (ng/ml) before exogenous progesterone administration	Post hoc test (p-value) Bonferroni	Multiple group test Kruskal Wallis
**108±5hrs vs 120±5hrs**	0.045	
**108±5hrs vs 144±5hrs**	0.389	0.6122
**120±5hrs vs 144±5hrs**	1	

Body mass index (BMI)	Post hoc test (p-value) Bonferroni	Multiple group test Kruskal Wallis
**108±5hrs vs 120±5hrs**	0.72	
**108±5hrs vs 144±5hrs**	0.69	0.4156
**120±5hrs vs 144±5hrs**	1	

Endometrial thickness (mm)	Post hoc test (p-value) Bonferroni	Multiple group test Kruskal Wallis
**108±5hrs vs 120±5hrs**	0.93	
**108±5hrs vs 144±5hrs**	1	0.3032
**120±5hrs vs 144±5hrs**	1	
