# Supplementary material for: Development of a Novel Endometrial Signature Based on Endometrial microRNA for Determining the Optimal Timing for Embryo Transfer
Source: Biomedicines. 2024 Mar 21;12(3):700. doi: 10.3390/biomedicines12030700 (PMC10968378; doi:10.3390/biomedicines12030700)
Supplement: Supplementary file 1 [file biomedicines-12-00700-s001.zip › Table S2.pdf]

Supplemental Table S2. A list of the reproductive disease-related miRNA-gene interactions

miRTarBase ID	miRNA	Target Gene	Function	References (PMID)
MIRT000071	hsa-miR-34a-5p	BIRC3	Reproductive Process	17540599
MIRT000135	hsa-miR-222-3p	SOD2	Development Process	19487542
MIRT000283	hsa-miR-15a-5p	MYB	Development Process	18818396
MIRT000283	hsa-miR-15a-5p	MYB	Development Process	21205891
MIRT000283	hsa-miR-15a-5p	MYB	Development Process	28337280
MIRT000331	hsa-miR-133a-3p	KRT7	Development Process	19378336
MIRT000340	hsa-miR-128-3p	NTRK3	Development Process	19370765
MIRT000340	hsa-miR-128-3p	NTRK3	Development Process	21143953
MIRT000365	hsa-miR-122-5p	SRF	Embryo Development	19726678
MIRT000497	hsa-miR-20b-5p	STAT3	Reproductive Process	20232316
MIRT000497	hsa-miR-20b-5p	STAT3	Reproductive Process	22473208
MIRT000640	hsa-miR-9-5p	BCL6	Reproductive Process	19956200
MIRT000720	hsa-miR-34a-5p	MYB	Development Process	19584398
MIRT000720	hsa-miR-34a-5p	MYB	Development Process	21566225
MIRT000720	hsa-miR-34a-5p	MYB	Development Process	24504520
MIRT000866	hsa-miR-15a-5p	HSPA1A	Cytokinesis	18362358
MIRT000935	hsa-miR-150-5p	MYB	Development Process	18667440
MIRT000935	hsa-miR-150-5p	MYB	Development Process	18539114
MIRT000935	hsa-miR-150-5p	MYB	Development Process	18814950
MIRT000935	hsa-miR-150-5p	MYB	Development Process	21590770
MIRT000935	hsa-miR-150-5p	MYB	Development Process	20603081
MIRT000935	hsa-miR-150-5p	MYB	Development Process	17923094
MIRT000935	hsa-miR-150-5p	MYB	Development Process	22025269
MIRT000935	hsa-miR-150-5p	MYB	Development Process	23604034
MIRT000935	hsa-miR-150-5p	MYB	Development Process	24613688
MIRT000935	hsa-miR-150-5p	MYB	Development Process	24438945
MIRT000935	hsa-miR-150-5p	MYB	Development Process	24086639
MIRT000935	hsa-miR-150-5p	MYB	Development Process	23521217
MIRT001037	hsa-miR-125b-5p	LIF	Embryo Development	19011087
MIRT001037	hsa-miR-125b-5p	LIF	Embryo Development	25472924
MIRT001039	hsa-miR-26a-5p	LIF	Embryo Development	19011087
MIRT001232	hsa-miR-34a-5p	AXIN2	Embryo Development	19336450
MIRT001353	hsa-miR-1-3p	NOTCH2	Development Process	18668040
MIRT001353	hsa-miR-1-3p	NOTCH2	Development Process	18668037
MIRT001437	hsa-miR-16-5p	NOTCH2	Development Process	18668040
MIRT001437	hsa-miR-16-5p	NOTCH2	Development Process	22473208
MIRT001437	hsa-miR-16-5p	NOTCH2	Development Process	23824327

MIRT001456	hsa-miR-16-5p	HSPA1A	Cytokinesis	18362358
MIRT001456	hsa-miR-16-5p	HSPA1A	Cytokinesis	18668040
MIRT001473	hsa-miR-16-5p	CDK5RAP1	Development Process	18668040
MIRT001498	hsa-miR-155-5p	SYNE2	Development Process	18668040
MIRT001556	hsa-miR-155-5p	CUL4B	Development Process	18668040
MIRT001757	hsa-miR-34a-5p	DLL1	Embryo Development	14697198
MIRT001757	hsa-miR-34a-5p	DLL1	Embryo Development	19461653
MIRT001757	hsa-miR-34a-5p	DLL1	Embryo Development	20144220
MIRT001757	hsa-miR-34a-5p	DLL1	Embryo Development	23327670
MIRT001843	hsa-miR-1-3p	HAND2	Embryo Development	21169019
MIRT001843	hsa-miR-1-3p	HAND2	Embryo Development	15951802
MIRT002402	hsa-miR-27a-3p	SP3	Embryo Development	18006846
MIRT002402	hsa-miR-27a-3p	SP3	Embryo Development	20382698
MIRT002532	hsa-miR-373-3p	PRC1	Cytokinesis	15685193
MIRT002758	hsa-miR-1-3p	AXL	Reproductive Process	15685193
MIRT002758	hsa-miR-1-3p	AXL	Reproductive Process	18668040
MIRT002765	hsa-miR-1-3p	EML4	Cytokinesis	15685193
MIRT002946	hsa-miR-15a-5p	DMTF1	Development Process	15131085
MIRT002946	hsa-miR-15a-5p	DMTF1	Development Process	22473208
MIRT002946	hsa-miR-15a-5p	DMTF1	Development Process	21572407
MIRT002953	hsa-miR-23a-3p	HES1	Embryo Development	12808467
MIRT002953	hsa-miR-23a-3p	HES1	Embryo Development	15066185
MIRT003108	hsa-miR-122-5p	SLC7A11	Development Process	19296470
MIRT003170	hsa-miR-210-3p	CBX1	Cytokinesis	19826008
MIRT003323	hsa-miR-9-5p	NTRK3	Development Process	17483472
MIRT003323	hsa-miR-9-5p	NTRK3	Development Process	19188439
MIRT003323	hsa-miR-9-5p	NTRK3	Development Process	18973228
MIRT003324	hsa-miR-199a-3p	KRT7	Development Process	19378336
MIRT003536	hsa-miR-19a-3p	NR4A2	Development Process	19885849
MIRT003537	hsa-miR-302d-3p	NR4A2	Development Process	19885849
MIRT003538	hsa-miR-372-3p	NR4A2	Development Process	19885849
MIRT003598	hsa-miR-200c-3p	EP300	Embryo Development	19569050
MIRT003599	hsa-miR-200b-3p	EP300	Embryo Development	19569050
MIRT003600	hsa-miR-429	EP300	Embryo Development	19569050
MIRT003601	hsa-miR-182-5p	EP300	Embryo Development	19569050
MIRT003609	hsa-miR-29a-3p	DKK1	Embryo Development	20551325
MIRT003754	hsa-miR-125b-5p	NTRK3	Development Process	17483472
MIRT003755	hsa-miR-125a-5p	NTRK3	Development Process	17483472
MIRT003755	hsa-miR-125a-5p	NTRK3	Development Process	19179615
MIRT003755	hsa-miR-125a-5p	NTRK3	Development Process	18973228

MIRT004007	hsa-miR-200a-3p	VCAM1	Development Process	17875710
MIRT004044	hsa-miR-29a-3p	PPM1D	Cytokinesis	21175813
MIRT004050	hsa-miR-129-5p	ETV6	Reproductive Process	19487295
MIRT004081	hsa-miR-126-3p	VCAM1	Development Process	18227515
MIRT004086	hsa-miR-101-3p	EED	Development Process	19008416
MIRT004183	hsa-miR-197-3p	AGR2	Development Process	16822819
MIRT004194	hsa-miR-197-3p	LRP4	Embryo Development	16822819
MIRT004194	hsa-miR-197-3p	LRP4	Embryo Development	23622248
MIRT004200	hsa-miR-197-3p	IER3	Development Process	16822819
MIRT004206	hsa-miR-197-3p	CES1	Development Process	16822819
MIRT004325	hsa-miR-199a-5p	LIF	Embryo Development	19011087
MIRT004325	hsa-miR-199a-5p	LIF	Embryo Development	19536157
MIRT004326	hsa-miR-125a-5p	LIF	Embryo Development	19011087
MIRT004425	hsa-miR-326	NOTCH2	Development Process	19955368
MIRT004463	hsa-miR-127-3p	BCL6	Reproductive Process	17581274
MIRT004463	hsa-miR-127-3p	BCL6	Reproductive Process	16766263
MIRT004463	hsa-miR-127-3p	BCL6	Reproductive Process	24282530
MIRT004520	hsa-let-7a-5p	IGF2	Embryo Development	17974952
MIRT004565	hsa-miR-299-5p	SPP1	Reproductive Process	19538464
MIRT004637	hsa-miR-16-5p	MYB	Development Process	21205891
MIRT004911	hsa-miR-198	NTRK3	Development Process	19370765
MIRT004914	hsa-miR-625-5p	NTRK3	Development Process	19370765
MIRT004936	hsa-miR-125b-5p	KRT7	Development Process	19378336
MIRT004937	hsa-miR-195-5p	KRT7	Development Process	19378336
MIRT004938	hsa-miR-145-5p	KRT7	Development Process	19378336
MIRT004984	hsa-miR-31-5p	FZD3	Embryo Development	19524507
MIRT005006	hsa-miR-125b-5p	STAT3	Reproductive Process	17891175
MIRT005006	hsa-miR-125b-5p	STAT3	Reproductive Process	28108732
MIRT005006	hsa-miR-125b-5p	STAT3	Reproductive Process	27576314
MIRT005029	hsa-miR-34a-5p	NOTCH2	Development Process	19773441
MIRT005029	hsa-miR-34a-5p	NOTCH2	Development Process	22363487
MIRT005089	hsa-miR-1-3p	TWF1	Cytokinesis	15685193
MIRT005089	hsa-miR-1-3p	TWF1	Cytokinesis	18668040
MIRT005089	hsa-miR-1-3p	TWF1	Cytokinesis	20571053
MIRT005125	hsa-miR-30a-5p	IDH1	Reproductive Process	18668040
MIRT005145	hsa-miR-30a-5p	SLC7A11	Development Process	18668040
MIRT005164	hsa-miR-30a-5p	TWF1	Cytokinesis	18668040
MIRT005164	hsa-miR-30a-5p	TWF1	Cytokinesis	22473208
MIRT005246	hsa-miR-155-5p	TWF1	Cytokinesis	18668040
MIRT005246	hsa-miR-155-5p	TWF1	Cytokinesis	20584899

MIRT005246	hsa-miR-155-5p	TWF1	Cytokinesis	22815788
MIRT005293	hsa-let-7a-5p	CASP8	Embryo Development	18758960
MIRT005360	hsa-miR-16-5p	PPM1D	Cytokinesis	20668064
MIRT005538	hsa-miR-126-3p	TWF1	Cytokinesis	20571053
MIRT005683	hsa-miR-145-5p	PPM1D	Cytokinesis	20668064
MIRT005684	hsa-miR-203a-3p	PPM1D	Cytokinesis	20668064
MIRT005738	hsa-miR-125b-5p	IGF2	Embryo Development	21200031
MIRT005739	hsa-miR-150-5p	IGF2	Embryo Development	21200031
MIRT005745	hsa-miR-17-3p	SOD2	Development Process	21203553
MIRT005745	hsa-miR-17-3p	SOD2	Development Process	27505139
MIRT005787	hsa-miR-221-3p	CORO1A	Cytokinesis	21226887
MIRT005791	hsa-miR-222-3p	CORO1A	Cytokinesis	21226887
MIRT005874	hsa-miR-31-5p	DKK1	Embryo Development	21048943
MIRT005884	hsa-miR-155-5p	MYB	Development Process	21062812
MIRT005884	hsa-miR-155-5p	MYB	Development Process	20680360
MIRT005884	hsa-miR-155-5p	MYB	Development Process	23807165
MIRT005962	hsa-miR-151a-3p	NTRK3	Development Process	21143953
MIRT006153	hsa-miR-483-5p	SRF	Embryo Development	21893058
MIRT006248	hsa-miR-223-3p	LIF	Embryo Development	22080513
MIRT006249	hsa-miR-223-3p	SP3	Embryo Development	22080513
MIRT006280	hsa-miR-23a-3p	PPARGC1A	Development Process	22318941
MIRT006324	hsa-miR-30b-5p	BCL6	Reproductive Process	22387553
MIRT006386	hsa-miR-29c-3p	IGFBP1	Development Process	21436257
MIRT006481	hsa-miR-181a-5p	DUSP6	Development Process	17382377
MIRT006481	hsa-miR-181a-5p	DUSP6	Development Process	25477247
MIRT006649	hsa-miR-409-3p	ANG	Reproductive Process	22531314
MIRT006664	hsa-miR-34a-5p	AXL	Reproductive Process	21814748
MIRT006664	hsa-miR-34a-5p	AXL	Reproductive Process	21566225
MIRT006664	hsa-miR-34a-5p	AXL	Reproductive Process	26667302
MIRT006664	hsa-miR-34a-5p	AXL	Reproductive Process	26287733
MIRT006750	hsa-miR-15b-5p	AXIN2	Embryo Development	21501592
MIRT006750	hsa-miR-15b-5p	AXIN2	Embryo Development	23446348
MIRT006750	hsa-miR-15b-5p	AXIN2	Embryo Development	21572407
MIRT006750	hsa-miR-15b-5p	AXIN2	Embryo Development	20371350
MIRT006751	hsa-miR-16-5p	AXIN2	Embryo Development	21501592
MIRT006751	hsa-miR-16-5p	AXIN2	Embryo Development	20371350
MIRT006751	hsa-miR-16-5p	AXIN2	Embryo Development	23446348
MIRT006751	hsa-miR-16-5p	AXIN2	Embryo Development	21572407
MIRT006833	hsa-miR-9-5p	SRF	Embryo Development	22907787
MIRT006834	hsa-miR-200a-3p	SRF	Embryo Development	22907787

MIRT006898	hsa-miR-23a-3p	FANCG	Reproductive Process	21750350
MIRT006927	hsa-miR-155-5p	BCL6	Reproductive Process	23041630
MIRT006927	hsa-miR-155-5p	BCL6	Reproductive Process	26986233
MIRT007067	hsa-miR-30b-5p	CAT	Development Process	22880027
MIRT007087	hsa-miR-150-5p	EP300	Embryo Development	23211718
MIRT007087	hsa-miR-150-5p	EP300	Embryo Development	27285761
MIRT007203	hsa-miR-30c-5p	TWF1	Cytokinesis	23224145
MIRT007203	hsa-miR-30c-5p	TWF1	Cytokinesis	22473208
MIRT016274	hsa-miR-193b-3p	SYNE2	Development Process	20304954
MIRT016311	hsa-miR-193b-3p	FANCI	Cytokinesis	20304954
MIRT016353	hsa-miR-193b-3p	NDC80	Cytokinesis	20304954
MIRT016365	hsa-miR-193b-3p	TMEM204	Development Process	20304954
MIRT016382	hsa-miR-193b-3p	KIF11	Cytokinesis	20304954
MIRT016385	hsa-miR-193b-3p	CDC20	Development Process	20304954
MIRT016386	hsa-miR-193b-3p	BUB1B	Reproductive Process	20304954
MIRT016386	hsa-miR-193b-3p	BUB1B	Reproductive Process	23622248
MIRT016405	hsa-miR-193b-3p	FANCG	Reproductive Process	20304954
MIRT016461	hsa-miR-193b-3p	SIX1	Embryo Development	20304954
MIRT016473	hsa-miR-193b-3p	TOP2A	Embryo Development	20304954
MIRT016475	hsa-miR-193b-3p	CBX1	Cytokinesis	20304954
MIRT016478	hsa-miR-193b-3p	ASPM	Reproductive Process	20304954
MIRT016532	hsa-miR-193b-3p	BORA	Cytokinesis	20304954
MIRT016551	hsa-miR-193b-3p	COTL1	Cytokinesis	21512034
MIRT016566	hsa-miR-193b-3p	TACC3	Development Process	20304954
MIRT016566	hsa-miR-193b-3p	TACC3	Development Process	23622248
MIRT016588	hsa-miR-193b-3p	MYB	Development Process	20304954
MIRT016588	hsa-miR-193b-3p	MYB	Development Process	25231743
MIRT016642	hsa-miR-429	RASSF2	Development Process	20005803
MIRT016724	hsa-miR-335-5p	EFNA1	Embryo Development	18185580
MIRT016866	hsa-miR-335-5p	SPP1	Reproductive Process	18185580
MIRT016961	hsa-miR-335-5p	HSPA1L	Reproductive Process	18185580
MIRT017025	hsa-miR-335-5p	SDCCAG8	Development Process	18185580
MIRT017044	hsa-miR-335-5p	THBD	Reproductive Process	18185580
MIRT017049	hsa-miR-335-5p	HEY1	Embryo Development	18185580
MIRT017134	hsa-miR-335-5p	DHRS3	Development Process	18185580
MIRT017152	hsa-miR-335-5p	MPPED2	Development Process	18185580
MIRT017160	hsa-miR-335-5p	SLC34A2	Embryo Development	18185580
MIRT017173	hsa-miR-335-5p	PLEKHA1	Reproductive Process	18185580
MIRT017228	hsa-miR-335-5p	SERPINF1	Reproductive Process	18185580
MIRT017345	hsa-miR-335-5p	PPARGC1A	Development Process	18185580

MIRT017395	hsa-miR-335-5p	CLDN4	Reproductive Process	18185580
MIRT017492	hsa-miR-335-5p	BCL6	Reproductive Process	18185580
MIRT017645	hsa-miR-335-5p	IDH1	Reproductive Process	18185580
MIRT017763	hsa-miR-335-5p	GABARAPL1	Cytokinesis	18185580
MIRT017798	hsa-miR-335-5p	GBP2	Cytokinesis	18185580
MIRT017844	hsa-miR-335-5p	DLL1	Embryo Development	18185580
MIRT017915	hsa-miR-335-5p	LRRC17	Development Process	18185580
MIRT017965	hsa-miR-335-5p	CATSPERB	Reproductive Process	18185580
MIRT018057	hsa-miR-335-5p	LIF	Embryo Development	18185580
MIRT018126	hsa-miR-335-5p	LMOD1	Development Process	18185580
MIRT018150	hsa-miR-335-5p	HSPA1A	Cytokinesis	18185580
MIRT018260	hsa-miR-335-5p	RICTOR	Embryo Development	18185580
MIRT018344	hsa-miR-335-5p	ANG	Reproductive Process	18185580
MIRT018347	hsa-miR-335-5p	DST	Cytokinesis	18185580
MIRT018401	hsa-miR-335-5p	AGR2	Development Process	18185580
MIRT018424	hsa-miR-335-5p	EVC	Development Process	18185580
MIRT018452	hsa-miR-335-5p	ID4	Reproductive Process	21618216
MIRT018452	hsa-miR-335-5p	ID4	Reproductive Process	20371350
MIRT018465	hsa-miR-335-5p	NR4A2	Development Process	18185580
MIRT018614	hsa-miR-335-5p	ANO1	Development Process	18185580
MIRT018619	hsa-miR-335-5p	EBF1	Development Process	18185580
MIRT018633	hsa-miR-335-5p	DUSP6	Development Process	18185580
MIRT018687	hsa-miR-335-5p	KLHL17	Development Process	18185580
MIRT018698	hsa-miR-335-5p	SPDEF	Development Process	18185580
MIRT018703	hsa-miR-335-5p	GDF15	Development Process	18185580
MIRT018847	hsa-miR-335-5p	SERPING1	Development Process	18185580
MIRT018916	hsa-miR-335-5p	CPE	Development Process	18185580
MIRT018982	hsa-miR-335-5p	WHRN	Development Process	18185580
MIRT019050	hsa-miR-335-5p	EIF2AK3	Development Process	18185580
MIRT019514	hsa-miR-151a-3p	MBTD1	Embryo Development	20371350
MIRT020112	hsa-miR-130b-3p	GAS1	Development Process	20371350
MIRT020290	hsa-miR-130b-3p	MPP5	Development Process	20371350
MIRT020309	hsa-miR-130b-3p	TRIM59	Cytokinesis	20371350
MIRT020352	hsa-miR-200a-3p	RASSF2	Development Process	20005803
MIRT020420	hsa-miR-106b-5p	OPRK1	Reproductive Process	17242205
MIRT020446	hsa-miR-106b-5p	SMAD9	Development Process	17242205
MIRT020611	hsa-miR-155-5p	CEP55	Development Process	18668040
MIRT020704	hsa-miR-155-5p	AIMP1	Development Process	18668040
MIRT020726	hsa-miR-155-5p	AXL	Reproductive Process	18668040
MIRT020789	hsa-miR-155-5p	GABARAPL1	Cytokinesis	20584899

MIRT020841	hsa-miR-155-5p	RICTOR	Embryo Development	18668040
MIRT020854	hsa-miR-155-5p	MPP5	Development Process	20584899
MIRT020854	hsa-miR-155-5p	MPP5	Development Process	22473208
MIRT020866	hsa-miR-155-5p	CAT	Development Process	18668040
MIRT020879	hsa-miR-155-5p	NOTCH2	Development Process	18668040
MIRT020896	hsa-miR-155-5p	SLC7A11	Development Process	18668040
MIRT020902	hsa-miR-155-5p	PSME4	Reproductive Process	18668040
MIRT020917	hsa-miR-155-5p	VCAM1	Development Process	21310411
MIRT020947	hsa-miR-155-5p	ALDH3A2	Development Process	18668040
MIRT021003	hsa-miR-155-5p	STAT3	Reproductive Process	18668040
MIRT021074	hsa-miR-200c-3p	RASSF2	Development Process	20005803
MIRT021209	hsa-miR-150-5p	PTPRR	Embryo Development	20218812
MIRT021244	hsa-miR-146a-5p	HSPA1A	Cytokinesis	18057241
MIRT021279	hsa-miR-146a-5p	SPP1	Reproductive Process	20110513
MIRT021293	hsa-miR-125a-5p	HOXC4	Embryo Development	20371350
MIRT021322	hsa-miR-9-5p	EFNA1	Embryo Development	17612493
MIRT021346	hsa-miR-9-5p	EP300	Embryo Development	17612493
MIRT021366	hsa-miR-9-5p	SYNE2	Development Process	17612493
MIRT021406	hsa-miR-9-5p	TAGLN	Development Process	17612493
MIRT021484	hsa-miR-9-5p	ID4	Reproductive Process	20371350
MIRT021510	hsa-miR-145-5p	NDRG2	Development Process	21351259
MIRT021513	hsa-miR-145-5p	MAP2K6	Reproductive Process	21351259
MIRT021645	hsa-miR-142-3p	PROM1	Development Process	21394831
MIRT021645	hsa-miR-142-3p	PROM1	Development Process	23619912
MIRT021654	hsa-miR-141-3p	RASSF2	Development Process	20005803
MIRT022008	hsa-miR-128-3p	MBTD1	Embryo Development	20371350
MIRT022013	hsa-miR-128-3p	ARID5B	Reproductive Process	20371350
MIRT022018	hsa-miR-128-3p	TAGLN	Development Process	17612493
MIRT022041	hsa-miR-128-3p	HEY2	Embryo Development	17612493
MIRT022074	hsa-miR-128-3p	SLC7A11	Development Process	17612493
MIRT023219	hsa-miR-122-5p	DZIP1L	Cytokinesis	19296470
MIRT023397	hsa-miR-122-5p	FOXK2	Development Process	17612493
MIRT023397	hsa-miR-122-5p	FOXK2	Development Process	23592263
MIRT023446	hsa-miR-30b-5p	EML4	Cytokinesis	20371350
MIRT023446	hsa-miR-30b-5p	EML4	Cytokinesis	23592263
MIRT023446	hsa-miR-30b-5p	EML4	Cytokinesis	21572407
MIRT023489	hsa-miR-1-3p	CUL4B	Development Process	18668040
MIRT023499	hsa-miR-1-3p	B3GNT2	Development Process	18668037
MIRT023519	hsa-miR-1-3p	FANCI	Cytokinesis	18668040
MIRT023547	hsa-miR-1-3p	SRF	Embryo Development	20458751

MIRT023596	hsa-miR-1-3p	SYNE2	Development Process	18668040
MIRT023613	hsa-miR-1-3p	DKK1	Embryo Development	18668037
MIRT023657	hsa-miR-1-3p	KLHL3	Development Process	18668037
MIRT023720	hsa-miR-1-3p	NR5A2	Embryo Development	18668037
MIRT023783	hsa-miR-1-3p	IQCD	Cytokinesis	18668037
MIRT023793	hsa-miR-1-3p	KIF4A	Cytokinesis	18668040
MIRT024011	hsa-miR-1-3p	COTL1	Cytokinesis	18668037
MIRT024131	hsa-miR-200b-3p	RASSF2	Development Process	20005803
MIRT024160	hsa-miR-221-3p	PTBP3	Development Process	20371350
MIRT024160	hsa-miR-221-3p	PTBP3	Development Process	21572407
MIRT024274	hsa-miR-215-5p	RAD54B	Reproductive Process	19074876
MIRT024294	hsa-miR-215-5p	THBD	Reproductive Process	19074876
MIRT024295	hsa-miR-215-5p	DIS3L	Cytokinesis	19074876
MIRT024324	hsa-miR-215-5p	CNEP1R1	Cytokinesis	19074876
MIRT024343	hsa-miR-215-5p	SS18	Development Process	19074876
MIRT024370	hsa-miR-215-5p	SLC7A11	Development Process	19074876
MIRT024373	hsa-miR-215-5p	TRIM59	Cytokinesis	19074876
MIRT024449	hsa-miR-215-5p	MYB	Development Process	19074876
MIRT024490	hsa-miR-215-5p	CENPE	Development Process	19074876
MIRT024587	hsa-miR-215-5p	SNX10	Development Process	19074876
MIRT024609	hsa-miR-215-5p	BUB1B	Reproductive Process	19074876
MIRT024617	hsa-miR-215-5p	ANG	Reproductive Process	19074876
MIRT024643	hsa-miR-215-5p	CDC20	Development Process	19074876
MIRT024665	hsa-miR-215-5p	DST	Cytokinesis	19074876
MIRT024686	hsa-miR-215-5p	ASPM	Reproductive Process	19074876
MIRT024697	hsa-miR-215-5p	LSM14B	Development Process	19074876
MIRT024718	hsa-miR-215-5p	KIF20A	Cytokinesis	19074876
MIRT024771	hsa-miR-215-5p	BORA	Cytokinesis	19074876
MIRT024792	hsa-miR-215-5p	DLGAP5	Cytokinesis	19074876
MIRT024828	hsa-miR-215-5p	CEP55	Development Process	19074876
MIRT024874	hsa-miR-215-5p	EIF2AK3	Development Process	19074876
MIRT024918	hsa-miR-215-5p	IL15	Development Process	19074876
MIRT024920	hsa-miR-215-5p	MNS1	Reproductive Process	19074876
MIRT025001	hsa-miR-183-5p	GAS1	Development Process	20371350
MIRT025026	hsa-miR-183-5p	NOTCH2	Development Process	20371350
MIRT025026	hsa-miR-183-5p	NOTCH2	Development Process	23824327
MIRT025039	hsa-miR-181a-5p	LRRC17	Development Process	17612493
MIRT025115	hsa-miR-181a-5p	H1F0	Cytokinesis	20371350
MIRT025115	hsa-miR-181a-5p	H1F0	Cytokinesis	26701625
MIRT025160	hsa-miR-181a-5p	HEY2	Embryo Development	17612493

MIRT025179	hsa-miR-181a-5p	SLC7A11	Development Process	17612493
MIRT025208	hsa-miR-181a-5p	NOTCH2	Development Process	20371350
MIRT025208	hsa-miR-181a-5p	NOTCH2	Development Process	22942087
MIRT025210	hsa-miR-34a-5p	KIF4A	Cytokinesis	21566225
MIRT025257	hsa-miR-34a-5p	GAS1	Development Process	20371350
MIRT025257	hsa-miR-34a-5p	GAS1	Development Process	23446348
MIRT025257	hsa-miR-34a-5p	GAS1	Development Process	21572407
MIRT025257	hsa-miR-34a-5p	GAS1	Development Process	24220341
MIRT025259	hsa-miR-34a-5p	RRAS	Development Process	20371350
MIRT025264	hsa-miR-34a-5p	SYNE2	Development Process	21566225
MIRT025295	hsa-miR-34a-5p	KIF11	Cytokinesis	21566225
MIRT025302	hsa-miR-34a-5p	HSPA1A	Cytokinesis	21566225
MIRT025331	hsa-miR-34a-5p	ANXA4	Development Process	21566225
MIRT025344	hsa-miR-34a-5p	ZFR	Development Process	21566225
MIRT025414	hsa-miR-34a-5p	CDC20	Development Process	21566225
MIRT025414	hsa-miR-34a-5p	CDC20	Development Process	23622248
MIRT025585	hsa-miR-34a-5p	CDKN2C	Development Process	21128241
MIRT025743	hsa-miR-7-5p	KRT7	Development Process	19073608
MIRT025870	hsa-miR-7-5p	MPP5	Development Process	20371350
MIRT025891	hsa-miR-7-5p	TAGLN	Development Process	17612493
MIRT025933	hsa-miR-7-5p	ALDH3A2	Development Process	19073608
MIRT026025	hsa-miR-198	MYB	Development Process	18667440
MIRT026028	hsa-miR-196a-5p	SRRT	Development Process	20371350
MIRT026028	hsa-miR-196a-5p	SRRT	Development Process	23622248
MIRT026028	hsa-miR-196a-5p	SRRT	Development Process	24398324
MIRT026028	hsa-miR-196a-5p	SRRT	Development Process	23446348
MIRT026028	hsa-miR-196a-5p	SRRT	Development Process	21572407
MIRT026117	hsa-miR-192-5p	FANCI	Cytokinesis	19074876
MIRT026159	hsa-miR-192-5p	THBD	Reproductive Process	19074876
MIRT026201	hsa-miR-192-5p	LSM14B	Development Process	19074876
MIRT026236	hsa-miR-192-5p	CENPE	Development Process	19074876
MIRT026262	hsa-miR-192-5p	CNEP1R1	Cytokinesis	19074876
MIRT026277	hsa-miR-192-5p	CEP55	Development Process	19074876
MIRT026319	hsa-miR-192-5p	CDC20	Development Process	19074876
MIRT026320	hsa-miR-192-5p	BUB1B	Reproductive Process	19074876
MIRT026419	hsa-miR-192-5p	DIS3L	Cytokinesis	19074876
MIRT026460	hsa-miR-192-5p	ASPM	Reproductive Process	19074876
MIRT026492	hsa-miR-192-5p	MNS1	Reproductive Process	19074876
MIRT026532	hsa-miR-192-5p	IL15	Development Process	19074876
MIRT026539	hsa-miR-192-5p	TRIM59	Cytokinesis	19074876

MIRT026610	hsa-miR-192-5p	ANG	Reproductive Process	19074876
MIRT026612	hsa-miR-192-5p	DST	Cytokinesis	19074876
MIRT026647	hsa-miR-192-5p	MPP5	Development Process	19074876
MIRT026660	hsa-miR-192-5p	SS18	Development Process	19074876
MIRT026724	hsa-miR-192-5p	SLC7A11	Development Process	19074876
MIRT026740	hsa-miR-192-5p	ID4	Reproductive Process	20371350
MIRT026783	hsa-miR-192-5p	SNX10	Development Process	19074876
MIRT026800	hsa-miR-192-5p	RAD54B	Reproductive Process	19074876
MIRT026817	hsa-miR-192-5p	EIF2AK3	Development Process	19074876
MIRT026832	hsa-miR-192-5p	DLGAP5	Cytokinesis	19074876
MIRT026859	hsa-miR-192-5p	KIF20A	Cytokinesis	19074876
MIRT026923	hsa-miR-192-5p	BORA	Cytokinesis	19074876
MIRT026947	hsa-miR-192-5p	MYB	Development Process	19074876
MIRT026998	hsa-miR-103a-3p	CEP55	Development Process	20371350
MIRT027072	hsa-miR-103a-3p	DMTF1	Development Process	20371350
MIRT027090	hsa-miR-103a-3p	GABARAPL1	Cytokinesis	20371350
MIRT027137	hsa-miR-103a-3p	AXIN2	Embryo Development	20371350
MIRT027137	hsa-miR-103a-3p	AXIN2	Embryo Development	23446348
MIRT027137	hsa-miR-103a-3p	AXIN2	Embryo Development	21572407
MIRT027302	hsa-miR-101-3p	ARID5B	Reproductive Process	20371350
MIRT027315	hsa-miR-101-3p	MBTD1	Embryo Development	20371350
MIRT028035	hsa-miR-93-5p	FIGNL1	Reproductive Process	20371350
MIRT028076	hsa-miR-93-5p	PLEKHA1	Reproductive Process	20371350
MIRT028178	hsa-miR-93-5p	STAT3	Reproductive Process	20371350
MIRT028178	hsa-miR-93-5p	STAT3	Reproductive Process	22473208
MIRT028409	hsa-miR-30a-5p	SOD2	Development Process	18668040
MIRT028518	hsa-miR-30a-5p	CDC20	Development Process	18668040
MIRT028535	hsa-miR-30a-5p	KIF11	Cytokinesis	18668040
MIRT028535	hsa-miR-30a-5p	KIF11	Cytokinesis	21572407
MIRT028535	hsa-miR-30a-5p	KIF11	Cytokinesis	20371350
MIRT028604	hsa-miR-30a-5p	PSME4	Reproductive Process	18668040
MIRT028614	hsa-miR-30a-5p	CAT	Development Process	18668040
MIRT028697	hsa-miR-27a-3p	ID4	Reproductive Process	20371350
MIRT028724	hsa-miR-27a-3p	MBTD1	Embryo Development	20371350
MIRT030477	hsa-miR-24-3p	SRRT	Development Process	17686970
MIRT030627	hsa-miR-24-3p	CORO1A	Cytokinesis	20138800
MIRT030642	hsa-miR-22-3p	ARID5B	Reproductive Process	20371350
MIRT030685	hsa-miR-21-5p	FANCI	Cytokinesis	18591254
MIRT030732	hsa-miR-21-5p	SYNE2	Development Process	18591254
MIRT030792	hsa-miR-21-5p	PLEKHA1	Reproductive Process	18591254

MIRT030806	hsa-miR-21-5p	DMTF1	Development Process	18591254
MIRT030890	hsa-miR-21-5p	PTBP3	Development Process	18591254
MIRT030898	hsa-miR-21-5p	TRIM59	Cytokinesis	18591254
MIRT030914	hsa-miR-21-5p	TOP2A	Embryo Development	18591254
MIRT030917	hsa-miR-21-5p	MPP5	Development Process	18591254
MIRT030942	hsa-miR-21-5p	ADGRG2	Reproductive Process	18591254
MIRT031079	hsa-miR-21-5p	STAT3	Reproductive Process	20048743
MIRT031079	hsa-miR-21-5p	STAT3	Reproductive Process	18591254
MIRT031079	hsa-miR-21-5p	STAT3	Reproductive Process	23998932
MIRT031079	hsa-miR-21-5p	STAT3	Reproductive Process	26549725
MIRT031165	hsa-miR-19b-3p	TRIM59	Cytokinesis	20371350
MIRT031279	hsa-miR-19b-3p	RASSF2	Development Process	20371350
MIRT031279	hsa-miR-19b-3p	RASSF2	Development Process	22473208
MIRT031510	hsa-miR-16-5p	CEP55	Development Process	20371350
MIRT031510	hsa-miR-16-5p	CEP55	Development Process	24398324
MIRT031510	hsa-miR-16-5p	CEP55	Development Process	23446348
MIRT031510	hsa-miR-16-5p	CEP55	Development Process	21572407
MIRT031610	hsa-miR-16-5p	AIMP1	Development Process	18668040
MIRT031615	hsa-miR-16-5p	CDC20	Development Process	18668040
MIRT031653	hsa-miR-16-5p	GABARAPL1	Cytokinesis	20371350
MIRT031653	hsa-miR-16-5p	GABARAPL1	Cytokinesis	23446348
MIRT031653	hsa-miR-16-5p	GABARAPL1	Cytokinesis	21572407
MIRT031727	hsa-miR-16-5p	DMTF1	Development Process	20371350
MIRT031727	hsa-miR-16-5p	DMTF1	Development Process	22473208
MIRT031727	hsa-miR-16-5p	DMTF1	Development Process	21572407
MIRT031760	hsa-miR-16-5p	CNOT7	Cytokinesis	18668040
MIRT031808	hsa-miR-16-5p	HSPH1	Cytokinesis	18668040
MIRT031835	hsa-miR-16-5p	PSME4	Reproductive Process	18668040
MIRT031894	hsa-miR-16-5p	EML4	Cytokinesis	18668040
MIRT031939	hsa-miR-16-5p	LAMB3	Embryo Development	18668040
MIRT031989	hsa-miR-16-5p	CASK	Cytokinesis	18668040
MIRT031989	hsa-miR-16-5p	CASK	Cytokinesis	23446348
MIRT031989	hsa-miR-16-5p	CASK	Cytokinesis	22927820
MIRT031989	hsa-miR-16-5p	CASK	Cytokinesis	27292025
MIRT031989	hsa-miR-16-5p	CASK	Cytokinesis	27418678
MIRT031989	hsa-miR-16-5p	CASK	Cytokinesis	28735896
MIRT032015	hsa-miR-16-5p	ARG2	Development Process	21199864
MIRT032381	hsa-let-7b-5p	ORC4	Cytokinesis	18668040
MIRT035524	hsa-miR-21-5p	BCL6	Reproductive Process	23416424
MIRT038243	hsa-miR-330-5p	LIF	Embryo Development	23622248

MIRT038935	hsa-miR-31-3p	RICTOR	Embryo Development	23622248
MIRT039520	hsa-miR-652-3p	EML4	Cytokinesis	23622248
MIRT041291	hsa-miR-193b-3p	TWF1	Cytokinesis	23622248
MIRT041373	hsa-miR-193b-3p	ALDH3A2	Development Process	23622248
MIRT041390	hsa-miR-193b-3p	HSPH1	Cytokinesis	23622248
MIRT041490	hsa-miR-193b-3p	EP300	Embryo Development	23622248
MIRT041506	hsa-miR-193b-3p	HSPA1L	Reproductive Process	23622248
MIRT041646	hsa-miR-484	SYNE2	Development Process	23622248
MIRT041752	hsa-miR-484	H1F0	Cytokinesis	23622248
MIRT041782	hsa-miR-484	PTBP3	Development Process	23622248
MIRT041870	hsa-miR-484	ACTA2	Development Process	23622248
MIRT041922	hsa-miR-484	ZFR	Development Process	23622248
MIRT041943	hsa-miR-484	SRF	Embryo Development	23622248
MIRT042022	hsa-miR-484	DST	Cytokinesis	23622248
MIRT042140	hsa-miR-484	SP3	Embryo Development	23622248
MIRT042343	hsa-miR-484	SORD	Reproductive Process	23622248
MIRT042423	hsa-miR-425-3p	PPARGC1A	Development Process	23622248
MIRT042482	hsa-miR-423-3p	H1F0	Cytokinesis	23622248
MIRT042569	hsa-miR-423-3p	MYB	Development Process	23622248
MIRT042674	hsa-miR-196b-5p	CKB	Development Process	23622248
MIRT043961	hsa-miR-378a-5p	PPARGC1A	Development Process	23622248
MIRT044195	hsa-miR-99b-5p	ETV6	Reproductive Process	23622248
MIRT044244	hsa-miR-29c-3p	PPM1D	Cytokinesis	23622248
MIRT044358	hsa-miR-106b-5p	CNOT7	Cytokinesis	23622248
MIRT044358	hsa-miR-106b-5p	CNOT7	Cytokinesis	22473208
MIRT044887	hsa-miR-193a-3p	LSM14B	Development Process	23622248
MIRT045708	hsa-miR-125a-5p	PRC1	Cytokinesis	23622248
MIRT045823	hsa-miR-140-5p	EP300	Embryo Development	23622248
MIRT045837	hsa-miR-133a-3p	NR4A2	Development Process	23622248
MIRT046026	hsa-miR-125b-5p	FAT1	Development Process	23622248
MIRT046057	hsa-miR-125b-5p	TEF	Development Process	23622248
MIRT046094	hsa-miR-125b-5p	LSM14B	Development Process	23622248
MIRT046126	hsa-miR-30b-5p	HEY1	Embryo Development	23622248
MIRT046150	hsa-miR-30b-5p	TRIM59	Cytokinesis	23622248
MIRT046601	hsa-miR-222-3p	FAT1	Development Process	23622248
MIRT046625	hsa-miR-222-3p	HES1	Embryo Development	23622248
MIRT046768	hsa-miR-222-3p	SRRT	Development Process	23622248
MIRT046771	hsa-miR-222-3p	KIF4A	Cytokinesis	23622248
MIRT046813	hsa-miR-222-3p	SS18	Development Process	23622248
MIRT047114	hsa-miR-183-5p	FOXK2	Development Process	23622248

MIRT047237	hsa-miR-181b-5p	CAT	Development Process	23622248
MIRT047254	hsa-miR-181b-5p	EP300	Embryo Development	23622248
MIRT047282	hsa-miR-181b-5p	DIDO1	Cytokinesis	23622248
MIRT047315	hsa-miR-181a-5p	FAT1	Development Process	23622248
MIRT047318	hsa-miR-181a-5p	LAMA3	Embryo Development	23622248
MIRT047336	hsa-miR-181a-5p	MTCL1	Cytokinesis	23622248
MIRT047352	hsa-miR-34a-5p	EXT2	Embryo Development	23622248
MIRT047657	hsa-miR-10a-5p	SORD	Reproductive Process	23622248
MIRT047766	hsa-miR-7-5p	TOP2A	Embryo Development	23622248
MIRT047921	hsa-miR-30c-5p	CSNK1E	Development Process	23622248
MIRT047948	hsa-miR-30c-5p	EP300	Embryo Development	23622248
MIRT047996	hsa-miR-30c-5p	PTBP3	Development Process	23622248
MIRT048010	hsa-miR-30c-5p	HSPH1	Cytokinesis	23622248
MIRT048018	hsa-miR-30c-5p	EED	Development Process	23622248
MIRT048018	hsa-miR-30c-5p	EED	Development Process	22473208
MIRT048100	hsa-miR-197-3p	ACTL9	Cytokinesis	23622248
MIRT048115	hsa-miR-197-3p	PTBP3	Development Process	23622248
MIRT048264	hsa-miR-196a-5p	NOTCH2	Development Process	23622248
MIRT048329	hsa-miR-106a-5p	NOTCH2	Development Process	23622248
MIRT048807	hsa-miR-93-5p	H1F0	Cytokinesis	23622248
MIRT048842	hsa-miR-93-5p	IGF2	Embryo Development	23622248
MIRT048962	hsa-miR-92a-3p	ETV6	Reproductive Process	23622248
MIRT049042	hsa-miR-92a-3p	DHRS3	Development Process	23622248
MIRT049047	hsa-miR-92a-3p	ADAMTS1	Reproductive Process	23622248
MIRT049124	hsa-miR-92a-3p	CKB	Development Process	23622248
MIRT049128	hsa-miR-92a-3p	CC2D2A	Embryo Development	23622248
MIRT049289	hsa-miR-92a-3p	DIDO1	Cytokinesis	23622248
MIRT049359	hsa-miR-92a-3p	STAT3	Reproductive Process	23622248
MIRT049359	hsa-miR-92a-3p	STAT3	Reproductive Process	23820254
MIRT049389	hsa-miR-92a-3p	CDC20	Development Process	23622248
MIRT049463	hsa-miR-92a-3p	HSPH1	Cytokinesis	23622248
MIRT049494	hsa-miR-92a-3p	HES1	Embryo Development	23622248
MIRT049535	hsa-miR-92a-3p	ASS1	Development Process	23622248
MIRT049540	hsa-miR-92a-3p	KIF20A	Cytokinesis	23622248
MIRT049544	hsa-miR-92a-3p	NOTCH2	Development Process	23622248
MIRT049550	hsa-miR-92a-3p	TRAF3IP1	Embryo Development	23622248
MIRT049644	hsa-miR-92a-3p	PRC1	Cytokinesis	23622248
MIRT049730	hsa-miR-92a-3p	SRRT	Development Process	23622248
MIRT049738	hsa-miR-92a-3p	KLHL3	Development Process	23622248
MIRT049743	hsa-miR-92a-3p	EP300	Embryo Development	23622248

MIRT049829	hsa-miR-92a-3p	SRF	Embryo Development	23622248
MIRT050017	hsa-miR-27a-3p	CEP55	Development Process	23622248
MIRT050193	hsa-miR-26a-5p	DST	Cytokinesis	23622248
MIRT050411	hsa-miR-23a-3p	KIF20A	Cytokinesis	23622248
MIRT050455	hsa-miR-22-3p	BUB1B	Reproductive Process	23622248
MIRT050559	hsa-miR-20a-5p	STAT3	Reproductive Process	23622248
MIRT050559	hsa-miR-20a-5p	STAT3	Reproductive Process	23836497
MIRT050559	hsa-miR-20a-5p	STAT3	Reproductive Process	22473208
MIRT050559	hsa-miR-20a-5p	STAT3	Reproductive Process	23059786
MIRT050668	hsa-miR-18a-5p	RICTOR	Embryo Development	23622248
MIRT050708	hsa-miR-18a-5p	CDC20	Development Process	23622248
MIRT050834	hsa-miR-17-5p	CPE	Development Process	23622248
MIRT050867	hsa-miR-17-5p	CNEP1R1	Cytokinesis	23622248
MIRT050901	hsa-miR-17-5p	OFD1	Development Process	23622248
MIRT050917	hsa-miR-17-5p	TSC2	Embryo Development	23622248
MIRT050936	hsa-miR-17-5p	NOTCH2	Development Process	23622248
MIRT051399	hsa-let-7f-5p	CNEP1R1	Cytokinesis	23622248
MIRT051400	hsa-let-7f-5p	CCNB2	Embryo Development	23622248
MIRT051849	hsa-let-7c-5p	FANCI	Cytokinesis	23622248
MIRT051850	hsa-let-7c-5p	NOTCH2	Development Process	23622248
MIRT051866	hsa-let-7c-5p	CCNB2	Embryo Development	23622248
MIRT051889	hsa-let-7b-5p	CCNB2	Embryo Development	23622248
MIRT051970	hsa-let-7b-5p	PPARGC1A	Development Process	23622248
MIRT051991	hsa-let-7b-5p	HES1	Embryo Development	23622248
MIRT051995	hsa-let-7b-5p	EP300	Embryo Development	23622248
MIRT052032	hsa-let-7b-5p	CEP126	Cytokinesis	23622248
MIRT052060	hsa-let-7b-5p	SYNE2	Development Process	23622248
MIRT052190	hsa-let-7b-5p	CKB	Development Process	23622248
MIRT052457	hsa-let-7a-5p	H1F0	Cytokinesis	23622248
MIRT052495	hsa-let-7a-5p	ASPM	Reproductive Process	23622248
MIRT052528	hsa-let-7a-5p	HOXC4	Embryo Development	23622248
MIRT052637	hsa-let-7a-5p	CCNB2	Embryo Development	23622248
MIRT052922	hsa-miR-200a-3p	MYB	Development Process	22101269
MIRT052923	hsa-miR-200b-3p	MYB	Development Process	22101269
MIRT052964	hsa-miR-200c-3p	MYB	Development Process	22101269
MIRT052965	hsa-miR-429	MYB	Development Process	22101269
MIRT052999	hsa-miR-100-5p	IGF2	Embryo Development	22926517
MIRT053039	hsa-miR-181a-5p	COL16A1	Reproductive Process	23238588
MIRT053071	hsa-miR-130b-3p	STAT3	Reproductive Process	24040078
MIRT053071	hsa-miR-130b-3p	STAT3	Reproductive Process	26475357

MIRT053156	hsa-miR-125a-5p	TEF	Development Process	24675842
MIRT053173	hsa-miR-21-5p	HPGD	Reproductive Process	24699315
MIRT053229	hsa-miR-106a-5p	STAT3	Reproductive Process	23836497
MIRT053229	hsa-miR-106a-5p	STAT3	Reproductive Process	23399684
MIRT053229	hsa-miR-106a-5p	STAT3	Reproductive Process	27325313
MIRT053299	hsa-miR-372-3p	DKK1	Embryo Development	22020335
MIRT053300	hsa-miR-373-3p	DKK1	Embryo Development	22020335
MIRT053300	hsa-miR-373-3p	DKK1	Embryo Development	28337453
MIRT053445	hsa-miR-203a-3p	SMAD9	Development Process	23807165
MIRT053497	hsa-miR-200c-3p	EFNA1	Embryo Development	23065816
MIRT053745	hsa-miR-29b-3p	MAP2K6	Reproductive Process	22942087
MIRT053748	hsa-miR-29b-3p	NOTCH2	Development Process	22942087
MIRT054144	hsa-miR-106b-5p	STAT3	Reproductive Process	25307786
MIRT054144	hsa-miR-106b-5p	STAT3	Reproductive Process	22473208
MIRT054144	hsa-miR-106b-5p	STAT3	Reproductive Process	26956882
MIRT054144	hsa-miR-106b-5p	STAT3	Reproductive Process	27325313
MIRT054155	hsa-miR-203a-3p	CASK	Cytokinesis	25373785
MIRT054186	hsa-miR-210-3p	PTBP3	Development Process	23024754
MIRT054244	hsa-miR-574-3p	EP300	Embryo Development	23554959
MIRT054262	hsa-miR-103a-3p	MYB	Development Process	25530421
MIRT054262	hsa-miR-103a-3p	MYB	Development Process	27888798
MIRT054375	hsa-miR-205-5p	BCL6	Reproductive Process	22870299
MIRT054414	hsa-miR-27a-3p	SLC7A11	Development Process	24516043
MIRT054582	hsa-miR-30b-5p	SIX1	Embryo Development	24593661
MIRT054810	hsa-miR-34a-5p	RICTOR	Embryo Development	24944883
MIRT054822	hsa-miR-30c-5p	IDH1	Reproductive Process	24623846
MIRT054849	hsa-miR-335-5p	SOX17	Embryo Development	24449834
MIRT054890	hsa-miR-195-5p	MYB	Development Process	24486218
MIRT054903	hsa-miR-17-5p	STAT3	Reproductive Process	25594054
MIRT054903	hsa-miR-17-5p	STAT3	Reproductive Process	22473208
MIRT054903	hsa-miR-17-5p	STAT3	Reproductive Process	23059786
MIRT055787	hsa-miR-92a-3p	PLEKHA1	Reproductive Process	23592263
MIRT055787	hsa-miR-92a-3p	PLEKHA1	Reproductive Process	24398324
MIRT055787	hsa-miR-92a-3p	PLEKHA1	Reproductive Process	23446348
MIRT055787	hsa-miR-92a-3p	PLEKHA1	Reproductive Process	22012620
MIRT055787	hsa-miR-92a-3p	PLEKHA1	Reproductive Process	21572407
MIRT055787	hsa-miR-92a-3p	PLEKHA1	Reproductive Process	20371350
MIRT055811	hsa-miR-15a-5p	PLEKHA1	Reproductive Process	21572407
MIRT055812	hsa-miR-16-5p	PLEKHA1	Reproductive Process	21572407
MIRT055813	hsa-miR-103a-3p	PLEKHA1	Reproductive Process	24398324

MIRT055813	hsa-miR-103a-3p	PLEKHA1	Reproductive Process	23446348
MIRT055813	hsa-miR-103a-3p	PLEKHA1	Reproductive Process	22012620
MIRT055813	hsa-miR-103a-3p	PLEKHA1	Reproductive Process	21572407
MIRT055813	hsa-miR-103a-3p	PLEKHA1	Reproductive Process	20371350
MIRT055816	hsa-miR-15b-5p	PLEKHA1	Reproductive Process	21572407
MIRT055817	hsa-miR-195-5p	PLEKHA1	Reproductive Process	21572407
MIRT056750	hsa-miR-196a-5p	ARID5B	Reproductive Process	23592263
MIRT056751	hsa-miR-196b-5p	ARID5B	Reproductive Process	23592263
MIRT057483	hsa-miR-30c-5p	KIF11	Cytokinesis	21572407
MIRT057483	hsa-miR-30c-5p	KIF11	Cytokinesis	20371350
MIRT057484	hsa-miR-30d-5p	KIF11	Cytokinesis	21572407
MIRT057484	hsa-miR-30d-5p	KIF11	Cytokinesis	20371350
MIRT057485	hsa-miR-30b-5p	KIF11	Cytokinesis	21572407
MIRT057485	hsa-miR-30b-5p	KIF11	Cytokinesis	20371350
MIRT057486	hsa-miR-30e-5p	KIF11	Cytokinesis	21572407
MIRT057486	hsa-miR-30e-5p	KIF11	Cytokinesis	20371350
MIRT057490	hsa-miR-19a-3p	CEP55	Development Process	21572407
MIRT057490	hsa-miR-19a-3p	CEP55	Development Process	20371350
MIRT057491	hsa-miR-19b-3p	CEP55	Development Process	21572407
MIRT057491	hsa-miR-19b-3p	CEP55	Development Process	20371350
MIRT057494	hsa-miR-130a-3p	CEP55	Development Process	21572407
MIRT057494	hsa-miR-130a-3p	CEP55	Development Process	20371350
MIRT057495	hsa-miR-152-3p	CEP55	Development Process	21572407
MIRT057495	hsa-miR-152-3p	CEP55	Development Process	20371350
MIRT057497	hsa-miR-130b-3p	CEP55	Development Process	21572407
MIRT057497	hsa-miR-130b-3p	CEP55	Development Process	20371350
MIRT057514	hsa-miR-15a-5p	CEP55	Development Process	24398324
MIRT057514	hsa-miR-15a-5p	CEP55	Development Process	23446348
MIRT057514	hsa-miR-15a-5p	CEP55	Development Process	21572407
MIRT057514	hsa-miR-15a-5p	CEP55	Development Process	20371350
MIRT057516	hsa-miR-15b-5p	CEP55	Development Process	24398324
MIRT057516	hsa-miR-15b-5p	CEP55	Development Process	23446348
MIRT057516	hsa-miR-15b-5p	CEP55	Development Process	21572407
MIRT057516	hsa-miR-15b-5p	CEP55	Development Process	20371350
MIRT057517	hsa-miR-195-5p	CEP55	Development Process	24398324
MIRT057517	hsa-miR-195-5p	CEP55	Development Process	23446348
MIRT057517	hsa-miR-195-5p	CEP55	Development Process	21572407
MIRT057517	hsa-miR-195-5p	CEP55	Development Process	20371350
MIRT059817	hsa-miR-4306	EFNA1	Embryo Development	23592263
MIRT059817	hsa-miR-4306	EFNA1	Embryo Development	26701625

MIRT074762	hsa-miR-92a-3p	CNEP1R1	Cytokinesis	22473208
MIRT074762	hsa-miR-92a-3p	CNEP1R1	Cytokinesis	21572407
MIRT079410	hsa-miR-17-5p	FOXK2	Development Process	22473208
MIRT079410	hsa-miR-17-5p	FOXK2	Development Process	23446348
MIRT079410	hsa-miR-17-5p	FOXK2	Development Process	21572407
MIRT079411	hsa-miR-20a-5p	FOXK2	Development Process	22473208
MIRT079411	hsa-miR-20a-5p	FOXK2	Development Process	23446348
MIRT079411	hsa-miR-20a-5p	FOXK2	Development Process	21572407
MIRT079412	hsa-miR-93-5p	FOXK2	Development Process	22473208
MIRT079412	hsa-miR-93-5p	FOXK2	Development Process	23446348
MIRT079412	hsa-miR-93-5p	FOXK2	Development Process	21572407
MIRT079413	hsa-miR-106a-5p	FOXK2	Development Process	23446348
MIRT079413	hsa-miR-106a-5p	FOXK2	Development Process	21572407
MIRT079414	hsa-miR-7-5p	FOXK2	Development Process	23446348
MIRT079414	hsa-miR-7-5p	FOXK2	Development Process	21572407
MIRT079415	hsa-miR-106b-5p	FOXK2	Development Process	22473208
MIRT079415	hsa-miR-106b-5p	FOXK2	Development Process	23446348
MIRT079415	hsa-miR-106b-5p	FOXK2	Development Process	21572407
MIRT079419	hsa-miR-302d-3p	FOXK2	Development Process	23446348
MIRT079419	hsa-miR-302d-3p	FOXK2	Development Process	21572407
MIRT079420	hsa-miR-372-3p	FOXK2	Development Process	23446348
MIRT079420	hsa-miR-372-3p	FOXK2	Development Process	21572407
MIRT079421	hsa-miR-373-3p	FOXK2	Development Process	23446348
MIRT079421	hsa-miR-373-3p	FOXK2	Development Process	21572407
MIRT079422	hsa-miR-20b-5p	FOXK2	Development Process	22473208
MIRT079422	hsa-miR-20b-5p	FOXK2	Development Process	23446348
MIRT079422	hsa-miR-20b-5p	FOXK2	Development Process	21572407
MIRT088679	hsa-miR-30a-5p	EML4	Cytokinesis	23592263
MIRT088679	hsa-miR-30a-5p	EML4	Cytokinesis	21572407
MIRT088680	hsa-miR-30c-5p	EML4	Cytokinesis	23592263
MIRT088680	hsa-miR-30c-5p	EML4	Cytokinesis	21572407
MIRT088681	hsa-miR-30d-5p	EML4	Cytokinesis	23592263
MIRT088681	hsa-miR-30d-5p	EML4	Cytokinesis	21572407
MIRT088682	hsa-miR-30e-5p	EML4	Cytokinesis	23592263
MIRT088682	hsa-miR-30e-5p	EML4	Cytokinesis	21572407
MIRT089105	hsa-miR-15a-5p	B3GNT2	Development Process	21572407
MIRT089105	hsa-miR-15a-5p	B3GNT2	Development Process	20371350
MIRT089106	hsa-miR-16-5p	B3GNT2	Development Process	21572407
MIRT089106	hsa-miR-16-5p	B3GNT2	Development Process	20371350
MIRT089108	hsa-miR-103a-3p	B3GNT2	Development Process	21572407

MIRT089108	hsa-miR-103a-3p	B3GNT2	Development Process	20371350
MIRT089110	hsa-miR-15b-5p	B3GNT2	Development Process	21572407
MIRT089110	hsa-miR-15b-5p	B3GNT2	Development Process	20371350
MIRT089111	hsa-miR-195-5p	B3GNT2	Development Process	21572407
MIRT089111	hsa-miR-195-5p	B3GNT2	Development Process	20371350
MIRT093076	hsa-miR-19a-3p	SLC7A11	Development Process	21572407
MIRT093077	hsa-miR-19b-3p	SLC7A11	Development Process	21572407
MIRT099596	hsa-miR-18a-5p	ID4	Reproductive Process	20371350
MIRT099597	hsa-miR-10a-5p	ID4	Reproductive Process	23446348
MIRT099597	hsa-miR-10a-5p	ID4	Reproductive Process	22012620
MIRT099597	hsa-miR-10a-5p	ID4	Reproductive Process	20371350
MIRT099598	hsa-miR-10b-5p	ID4	Reproductive Process	23446348
MIRT099598	hsa-miR-10b-5p	ID4	Reproductive Process	22012620
MIRT099598	hsa-miR-10b-5p	ID4	Reproductive Process	20371350
MIRT099599	hsa-miR-18b-5p	ID4	Reproductive Process	20371350
MIRT099600	hsa-miR-486-5p	ID4	Reproductive Process	23446348
MIRT099600	hsa-miR-486-5p	ID4	Reproductive Process	22012620
MIRT099600	hsa-miR-486-5p	ID4	Reproductive Process	20371350
MIRT100353	hsa-miR-223-5p	HSPA1A	Cytokinesis	22012620
MIRT102166	hsa-miR-196b-5p	SRRT	Development Process	24398324
MIRT102166	hsa-miR-196b-5p	SRRT	Development Process	23446348
MIRT102166	hsa-miR-196b-5p	SRRT	Development Process	21572407
MIRT102166	hsa-miR-196b-5p	SRRT	Development Process	20371350
MIRT103433	hsa-miR-92a-3p	SNX10	Development Process	22473208
MIRT104234	hsa-miR-15b-5p	DMTF1	Development Process	22473208
MIRT104234	hsa-miR-15b-5p	DMTF1	Development Process	21572407
MIRT104235	hsa-miR-195-5p	DMTF1	Development Process	22473208
MIRT104235	hsa-miR-195-5p	DMTF1	Development Process	21572407
MIRT106868	hsa-miR-181a-5p	PTBP3	Development Process	23824327
MIRT106869	hsa-miR-181b-5p	PTBP3	Development Process	23824327
MIRT138701	hsa-miR-302d-3p	MPP5	Development Process	22012620
MIRT138702	hsa-miR-372-3p	MPP5	Development Process	22012620
MIRT138703	hsa-miR-373-3p	MPP5	Development Process	22012620
MIRT145857	hsa-miR-200a-3p	STAT3	Reproductive Process	26535690
MIRT154043	hsa-miR-15a-5p	RASSF2	Development Process	23708386
MIRT154044	hsa-miR-16-5p	RASSF2	Development Process	23708386
MIRT154048	hsa-miR-15b-5p	RASSF2	Development Process	23708386
MIRT154051	hsa-miR-195-5p	RASSF2	Development Process	23708386
MIRT171395	hsa-miR-155-5p	DMTF1	Development Process	22473208
MIRT172594	hsa-miR-19a-3p	CNOT7	Cytokinesis	22473208

MIRT172596	hsa-miR-19b-3p	CNOT7	Cytokinesis	22473208
MIRT180171	hsa-miR-182-5p	NOTCH2	Development Process	23824327
MIRT186534	hsa-miR-92a-3p	TWF1	Cytokinesis	23592263
MIRT186534	hsa-miR-92a-3p	TWF1	Cytokinesis	20371350
MIRT197720	hsa-miR-15b-5p	PPM1D	Cytokinesis	25092292
MIRT204830	hsa-miR-106b-5p	CASP8	Embryo Development	24166509
MIRT206479	hsa-miR-142-3p	EML4	Cytokinesis	22473208
MIRT242416	hsa-miR-92a-3p	CCDC113	Cytokinesis	22473208
MIRT242416	hsa-miR-92a-3p	CCDC113	Cytokinesis	23824327
MIRT245473	hsa-miR-4306	ARID5B	Reproductive Process	23313552
MIRT247368	hsa-miR-15a-5p	GABARAPL1	Cytokinesis	23446348
MIRT247368	hsa-miR-15a-5p	GABARAPL1	Cytokinesis	21572407
MIRT247368	hsa-miR-15a-5p	GABARAPL1	Cytokinesis	20371350
MIRT247369	hsa-miR-15b-5p	GABARAPL1	Cytokinesis	23446348
MIRT247369	hsa-miR-15b-5p	GABARAPL1	Cytokinesis	21572407
MIRT247369	hsa-miR-15b-5p	GABARAPL1	Cytokinesis	20371350
MIRT247370	hsa-miR-195-5p	GABARAPL1	Cytokinesis	23446348
MIRT247370	hsa-miR-195-5p	GABARAPL1	Cytokinesis	21572407
MIRT247370	hsa-miR-195-5p	GABARAPL1	Cytokinesis	20371350
MIRT257371	hsa-miR-181a-5p	ID4	Reproductive Process	20371350
MIRT257372	hsa-miR-181b-5p	ID4	Reproductive Process	20371350
MIRT270030	hsa-miR-143-3p	GABARAPL1	Cytokinesis	26349981
MIRT279714	hsa-let-7a-5p	ARG2	Development Process	26989813
MIRT284089	hsa-miR-199a-3p	CNEP1R1	Cytokinesis	23313552
MIRT301709	hsa-miR-92a-3p	TEF	Development Process	20371350
MIRT309648	hsa-miR-129-5p	CPE	Development Process	23824327
MIRT309648	hsa-miR-129-5p	CPE	Development Process	27418678
MIRT317106	hsa-miR-223-5p	ID4	Reproductive Process	20371350
MIRT405809	hsa-miR-129-5p	SIX1	Embryo Development	23824327
MIRT437348	hsa-let-7c-5p	RICTOR	Embryo Development	25311506
MIRT437349	hsa-miR-16-5p	RICTOR	Embryo Development	25311506
MIRT437377	hsa-miR-125b-5p	ENPEP	Development Process	24098452
MIRT437478	hsa-miR-663a	MYL9	Cytokinesis	24014830
MIRT437606	hsa-miR-142-3p	TWF1	Cytokinesis	23592263
MIRT437606	hsa-miR-142-3p	TWF1	Cytokinesis	20371350
MIRT437606	hsa-miR-142-3p	TWF1	Cytokinesis	22815788
MIRT437668	hsa-miR-214-3p	ABLIM3	Cytokinesis	22815788
MIRT437675	hsa-miR-214-3p	TWF1	Cytokinesis	22815788
MIRT437690	hsa-miR-223-3p	TWF1	Cytokinesis	22815788
MIRT437721	hsa-miR-29a-3p	AMFR	Development Process	22815788

MIRT437722	hsa-miR-29b-3p	AMFR	Development Process	22815788
MIRT437723	hsa-miR-29c-3p	AMFR	Development Process	22815788
MIRT437777	hsa-miR-1-3p	TH	Embryo Development	25512392
MIRT437882	hsa-miR-17-5p	CLU	Development Process	23436804
MIRT437939	hsa-miR-34a-5p	ARHGDIB	Development Process	23349340
MIRT438217	hsa-miR-181a-5p	STAT3	Reproductive Process	24329418
MIRT438224	hsa-miR-21-5p	CLU	Development Process	24327270
MIRT438421	hsa-miR-24-3p	TACC3	Development Process	23174883
MIRT438422	hsa-miR-152-3p	TACC3	Development Process	23174883
MIRT438552	hsa-miR-130b-3p	PPARGC1A	Development Process	23868745
MIRT438607	hsa-miR-125b-5p	DUSP6	Development Process	25001178
MIRT438639	hsa-miR-122-5p	AXL	Reproductive Process	24113455
MIRT439144	hsa-let-7c-5p	SPDEF	Development Process	25074381
MIRT439371	hsa-miR-106b-5p	TWF1	Cytokinesis	22473208
MIRT439372	hsa-miR-20a-5p	TWF1	Cytokinesis	22473208
MIRT439540	hsa-miR-142-3p	SS18	Development Process	22473208
MIRT439564	hsa-miR-382-5p	MTCL1	Cytokinesis	24374217
MIRT439566	hsa-miR-20a-5p	SOD2	Development Process	22473208
MIRT439566	hsa-miR-20a-5p	SOD2	Development Process	22012620
MIRT439567	hsa-miR-106b-5p	SOD2	Development Process	22473208
MIRT439567	hsa-miR-106b-5p	SOD2	Development Process	22012620
MIRT439593	hsa-miR-142-3p	SLC7A11	Development Process	22473208
MIRT439769	hsa-miR-142-3p	RICTOR	Embryo Development	22473208
MIRT440100	hsa-miR-205-5p	NOTCH2	Development Process	22473208
MIRT440368	hsa-miR-155-5p	KIF3A	Development Process	22473208
MIRT440468	hsa-miR-106b-5p	IER3	Development Process	22473208
MIRT440469	hsa-miR-20a-5p	IER3	Development Process	22473208
MIRT440866	hsa-miR-210-3p	CSNK1E	Development Process	22473208
MIRT440918	hsa-miR-20a-5p	CNOT7	Cytokinesis	22473208
MIRT442592	hsa-miR-203a-3p	SIX1	Embryo Development	22100165
MIRT442903	hsa-miR-199a-3p	SOD2	Development Process	22100165
MIRT451868	hsa-miR-4306	SOD2	Development Process	23592263
MIRT451868	hsa-miR-4306	SOD2	Development Process	24398324
MIRT451868	hsa-miR-4306	SOD2	Development Process	23446348
MIRT451868	hsa-miR-4306	SOD2	Development Process	21572407
MIRT451876	hsa-miR-539-5p	SOD2	Development Process	23592263
MIRT451876	hsa-miR-539-5p	SOD2	Development Process	24398324
MIRT451876	hsa-miR-539-5p	SOD2	Development Process	23446348
MIRT451876	hsa-miR-539-5p	SOD2	Development Process	21572407
MIRT453742	hsa-miR-4306	CSNK1E	Development Process	23592263

MIRT453751	hsa-miR-195-5p	CSNK1E	Development Process	23592263
MIRT453752	hsa-miR-16-5p	CSNK1E	Development Process	23592263
MIRT453753	hsa-miR-15b-5p	CSNK1E	Development Process	23592263
MIRT453754	hsa-miR-15a-5p	CSNK1E	Development Process	23592263
MIRT454267	hsa-miR-4772-3p	PSMA1	Cytokinesis	23592263
MIRT469208	hsa-miR-625-5p	RICTOR	Embryo Development	23592263
MIRT470910	hsa-miR-411-5p	PLEKHA1	Reproductive Process	23592263
MIRT470910	hsa-miR-411-5p	PLEKHA1	Reproductive Process	24398324
MIRT470910	hsa-miR-411-5p	PLEKHA1	Reproductive Process	23446348
MIRT470910	hsa-miR-411-5p	PLEKHA1	Reproductive Process	22012620
MIRT470910	hsa-miR-411-5p	PLEKHA1	Reproductive Process	21572407
MIRT470910	hsa-miR-411-5p	PLEKHA1	Reproductive Process	20371350
MIRT473845	hsa-miR-411-5p	MAP2K6	Reproductive Process	23592263
MIRT476749	hsa-miR-199a-3p	FOXK2	Development Process	23592263
MIRT476756	hsa-miR-203a-3p	FOXK2	Development Process	23592263
MIRT477653	hsa-miR-940	EFNA1	Embryo Development	23592263
MIRT478677	hsa-miR-142-3p	CSRP2	Development Process	23592263
MIRT478677	hsa-miR-142-3p	CSRP2	Development Process	21572407
MIRT478678	hsa-miR-155-5p	CSRP2	Development Process	23592263
MIRT478678	hsa-miR-155-5p	CSRP2	Development Process	21572407
MIRT478692	hsa-miR-27a-3p	CSRP2	Development Process	23592263
MIRT478692	hsa-miR-27a-3p	CSRP2	Development Process	21572407
MIRT478695	hsa-miR-128-3p	CSRP2	Development Process	23592263
MIRT478695	hsa-miR-128-3p	CSRP2	Development Process	21572407
MIRT478698	hsa-miR-199a-3p	CSRP2	Development Process	23592263
MIRT478698	hsa-miR-199a-3p	CSRP2	Development Process	21572407
MIRT481095	hsa-miR-24-3p	B3GNT2	Development Process	23592263
MIRT488649	hsa-miR-483-5p	HAND2	Embryo Development	23592263
MIRT489752	hsa-miR-663a	TACC3	Development Process	23592263
MIRT493009	hsa-miR-423-5p	NANOS1	Development Process	23592263
MIRT496595	hsa-miR-423-5p	TAGLN	Development Process	22291592
MIRT498666	hsa-miR-197-3p	SOD2	Development Process	24398324
MIRT498666	hsa-miR-197-3p	SOD2	Development Process	22012620
MIRT498670	hsa-miR-203a-3p	SOD2	Development Process	24398324
MIRT498670	hsa-miR-203a-3p	SOD2	Development Process	22012620
MIRT498972	hsa-miR-103a-3p	ORC4	Cytokinesis	24398324
MIRT498972	hsa-miR-103a-3p	ORC4	Cytokinesis	23446348
MIRT498972	hsa-miR-103a-3p	ORC4	Cytokinesis	21572407
MIRT498972	hsa-miR-103a-3p	ORC4	Cytokinesis	20371350
MIRT498972	hsa-miR-103a-3p	ORC4	Cytokinesis	27292025

MIRT498981	hsa-miR-195-5p	ORC4	Cytokinesis	24398324
MIRT498981	hsa-miR-195-5p	ORC4	Cytokinesis	23446348
MIRT498981	hsa-miR-195-5p	ORC4	Cytokinesis	21572407
MIRT498981	hsa-miR-195-5p	ORC4	Cytokinesis	20371350
MIRT498981	hsa-miR-195-5p	ORC4	Cytokinesis	27292025
MIRT498982	hsa-miR-16-5p	ORC4	Cytokinesis	24398324
MIRT498982	hsa-miR-16-5p	ORC4	Cytokinesis	23446348
MIRT498982	hsa-miR-16-5p	ORC4	Cytokinesis	21572407
MIRT498982	hsa-miR-16-5p	ORC4	Cytokinesis	20371350
MIRT498982	hsa-miR-16-5p	ORC4	Cytokinesis	27292025
MIRT498983	hsa-miR-15b-5p	ORC4	Cytokinesis	24398324
MIRT498983	hsa-miR-15b-5p	ORC4	Cytokinesis	23446348
MIRT498983	hsa-miR-15b-5p	ORC4	Cytokinesis	21572407
MIRT498983	hsa-miR-15b-5p	ORC4	Cytokinesis	20371350
MIRT498983	hsa-miR-15b-5p	ORC4	Cytokinesis	27292025
MIRT498984	hsa-miR-15a-5p	ORC4	Cytokinesis	24398324
MIRT498984	hsa-miR-15a-5p	ORC4	Cytokinesis	23446348
MIRT498984	hsa-miR-15a-5p	ORC4	Cytokinesis	21572407
MIRT498984	hsa-miR-15a-5p	ORC4	Cytokinesis	20371350
MIRT498984	hsa-miR-15a-5p	ORC4	Cytokinesis	27292025
MIRT499576	hsa-miR-31-5p	INTU	Embryo Development	24398324
MIRT499587	hsa-miR-7-5p	INTU	Embryo Development	24398324
MIRT499587	hsa-miR-7-5p	INTU	Embryo Development	20371350
MIRT504352	hsa-miR-23a-3p	VCAM1	Development Process	23446348
MIRT504352	hsa-miR-23a-3p	VCAM1	Development Process	21572407
MIRT504352	hsa-miR-23a-3p	VCAM1	Development Process	23824327
MIRT504352	hsa-miR-23a-3p	VCAM1	Development Process	23313552
MIRT504464	hsa-miR-145-5p	EID2B	Development Process	23446348
MIRT504466	hsa-miR-205-5p	EID2B	Development Process	23446348
MIRT504470	hsa-miR-92a-3p	EID2B	Development Process	22473208
MIRT504470	hsa-miR-92a-3p	EID2B	Development Process	23446348
MIRT508038	hsa-miR-195-5p	AXIN2	Embryo Development	23446348
MIRT508038	hsa-miR-195-5p	AXIN2	Embryo Development	21572407
MIRT508038	hsa-miR-195-5p	AXIN2	Embryo Development	20371350
MIRT508041	hsa-miR-15a-5p	AXIN2	Embryo Development	23446348
MIRT508041	hsa-miR-15a-5p	AXIN2	Embryo Development	21572407
MIRT508041	hsa-miR-15a-5p	AXIN2	Embryo Development	20371350
MIRT508642	hsa-miR-195-5p	CASK	Cytokinesis	23446348
MIRT508642	hsa-miR-195-5p	CASK	Cytokinesis	22927820
MIRT508642	hsa-miR-195-5p	CASK	Cytokinesis	27292025

MIRT508642	hsa-miR-195-5p	CASK	Cytokinesis	27418678
MIRT508642	hsa-miR-195-5p	CASK	Cytokinesis	28735896
MIRT508643	hsa-miR-15b-5p	CASK	Cytokinesis	23446348
MIRT508643	hsa-miR-15b-5p	CASK	Cytokinesis	22927820
MIRT508643	hsa-miR-15b-5p	CASK	Cytokinesis	27292025
MIRT508643	hsa-miR-15b-5p	CASK	Cytokinesis	27418678
MIRT508643	hsa-miR-15b-5p	CASK	Cytokinesis	28735896
MIRT508644	hsa-miR-15a-5p	CASK	Cytokinesis	23446348
MIRT508644	hsa-miR-15a-5p	CASK	Cytokinesis	22927820
MIRT508644	hsa-miR-15a-5p	CASK	Cytokinesis	27292025
MIRT508644	hsa-miR-15a-5p	CASK	Cytokinesis	27418678
MIRT508644	hsa-miR-15a-5p	CASK	Cytokinesis	28735896
MIRT509521	hsa-miR-151a-3p	INTU	Embryo Development	23446348
MIRT509521	hsa-miR-151a-3p	INTU	Embryo Development	22012620
MIRT509521	hsa-miR-151a-3p	INTU	Embryo Development	21572407
MIRT509521	hsa-miR-151a-3p	INTU	Embryo Development	20371350
MIRT511895	hsa-miR-152-3p	GAS1	Development Process	23446348
MIRT511895	hsa-miR-152-3p	GAS1	Development Process	21572407
MIRT511895	hsa-miR-152-3p	GAS1	Development Process	20371350
MIRT515266	hsa-miR-197-3p	CSNK1E	Development Process	23446348
MIRT515268	hsa-miR-411-5p	CSNK1E	Development Process	23446348
MIRT515921	hsa-miR-373-3p	MFAP2	Embryo Development	23446348
MIRT515921	hsa-miR-373-3p	MFAP2	Embryo Development	23824327
MIRT515922	hsa-miR-372-3p	MFAP2	Embryo Development	23446348
MIRT515922	hsa-miR-372-3p	MFAP2	Embryo Development	23824327
MIRT515924	hsa-miR-302d-3p	MFAP2	Embryo Development	23446348
MIRT515924	hsa-miR-302d-3p	MFAP2	Embryo Development	23824327
MIRT517270	hsa-miR-574-5p	ANG	Reproductive Process	23446348
MIRT517270	hsa-miR-574-5p	ANG	Reproductive Process	21572407
MIRT517271	hsa-miR-223-5p	ANG	Reproductive Process	23446348
MIRT517271	hsa-miR-223-5p	ANG	Reproductive Process	21572407
MIRT517946	hsa-miR-663a	TRIM59	Cytokinesis	23446348
MIRT522973	hsa-miR-150-5p	INTU	Embryo Development	23446348
MIRT523846	hsa-miR-574-5p	ETV6	Reproductive Process	23446348
MIRT523849	hsa-miR-223-5p	ETV6	Reproductive Process	23446348
MIRT525720	hsa-miR-202-3p	SOD2	Development Process	22012620
MIRT525725	hsa-let-7g-5p	SOD2	Development Process	22012620
MIRT525726	hsa-let-7f-5p	SOD2	Development Process	22012620
MIRT525728	hsa-let-7d-5p	SOD2	Development Process	22012620
MIRT525729	hsa-let-7c-5p	SOD2	Development Process	22012620

MIRT525730	hsa-let-7b-5p	SOD2	Development Process	22012620
MIRT525732	hsa-let-7a-5p	SOD2	Development Process	22012620
MIRT525736	hsa-miR-518a-5p	SOD2	Development Process	22012620
MIRT525762	hsa-miR-93-5p	SOD2	Development Process	22473208
MIRT525762	hsa-miR-93-5p	SOD2	Development Process	22012620
MIRT525765	hsa-miR-20b-5p	SOD2	Development Process	22473208
MIRT525765	hsa-miR-20b-5p	SOD2	Development Process	22012620
MIRT525766	hsa-miR-17-5p	SOD2	Development Process	22473208
MIRT525766	hsa-miR-17-5p	SOD2	Development Process	22012620
MIRT525767	hsa-miR-106a-5p	SOD2	Development Process	22012620
MIRT525811	hsa-miR-885-5p	SOD2	Development Process	22012620
MIRT527541	hsa-miR-373-3p	DLGAP5	Cytokinesis	22012620
MIRT527541	hsa-miR-373-3p	DLGAP5	Cytokinesis	21572407
MIRT527542	hsa-miR-372-3p	DLGAP5	Cytokinesis	22012620
MIRT527542	hsa-miR-372-3p	DLGAP5	Cytokinesis	21572407
MIRT527544	hsa-miR-302d-3p	DLGAP5	Cytokinesis	22012620
MIRT527544	hsa-miR-302d-3p	DLGAP5	Cytokinesis	21572407
MIRT527646	hsa-miR-21-5p	ORC4	Cytokinesis	22012620
MIRT527674	hsa-miR-29c-3p	CASP8	Embryo Development	22012620
MIRT527675	hsa-miR-29b-3p	CASP8	Embryo Development	22012620
MIRT527676	hsa-miR-29a-3p	CASP8	Embryo Development	22012620
MIRT528382	hsa-miR-205-5p	TRAF3IP1	Embryo Development	22012620
MIRT534192	hsa-miR-92a-3p	SLC7A11	Development Process	22473208
MIRT534192	hsa-miR-92a-3p	SLC7A11	Development Process	22012620
MIRT534196	hsa-miR-223-5p	SLC7A11	Development Process	22012620
MIRT534196	hsa-miR-223-5p	SLC7A11	Development Process	20371350
MIRT534196	hsa-miR-223-5p	SLC7A11	Development Process	23313552
MIRT534196	hsa-miR-223-5p	SLC7A11	Development Process	21572407
MIRT536445	hsa-miR-93-5p	KMT2B	Reproductive Process	22012620
MIRT536445	hsa-miR-93-5p	KMT2B	Reproductive Process	21572407
MIRT536445	hsa-miR-93-5p	KMT2B	Reproductive Process	20371350
MIRT536446	hsa-miR-20a-5p	KMT2B	Reproductive Process	22012620
MIRT536446	hsa-miR-20a-5p	KMT2B	Reproductive Process	21572407
MIRT536446	hsa-miR-20a-5p	KMT2B	Reproductive Process	20371350
MIRT536447	hsa-miR-17-5p	KMT2B	Reproductive Process	22012620
MIRT536447	hsa-miR-17-5p	KMT2B	Reproductive Process	21572407
MIRT536447	hsa-miR-17-5p	KMT2B	Reproductive Process	20371350
MIRT536448	hsa-miR-106b-5p	KMT2B	Reproductive Process	22012620
MIRT536448	hsa-miR-106b-5p	KMT2B	Reproductive Process	21572407
MIRT536448	hsa-miR-106b-5p	KMT2B	Reproductive Process	20371350

MIRT536449	hsa-miR-106a-5p	KMT2B	Reproductive Process	22012620
MIRT536449	hsa-miR-106a-5p	KMT2B	Reproductive Process	21572407
MIRT536449	hsa-miR-106a-5p	KMT2B	Reproductive Process	20371350
MIRT536450	hsa-miR-20b-5p	KMT2B	Reproductive Process	22012620
MIRT536450	hsa-miR-20b-5p	KMT2B	Reproductive Process	21572407
MIRT536450	hsa-miR-20b-5p	KMT2B	Reproductive Process	20371350
MIRT536454	hsa-miR-382-5p	KLHL3	Development Process	22012620
MIRT537930	hsa-miR-103a-3p	DST	Cytokinesis	22012620
MIRT542124	hsa-miR-150-5p	DIS3L	Cytokinesis	21572407
MIRT542134	hsa-miR-373-3p	DIS3L	Cytokinesis	21572407
MIRT542134	hsa-miR-373-3p	DIS3L	Cytokinesis	27292025
MIRT542135	hsa-miR-372-3p	DIS3L	Cytokinesis	21572407
MIRT542135	hsa-miR-372-3p	DIS3L	Cytokinesis	27292025
MIRT542137	hsa-miR-302d-3p	DIS3L	Cytokinesis	21572407
MIRT542137	hsa-miR-302d-3p	DIS3L	Cytokinesis	27292025
MIRT542141	hsa-miR-93-5p	DIS3L	Cytokinesis	21572407
MIRT542141	hsa-miR-93-5p	DIS3L	Cytokinesis	27292025
MIRT542144	hsa-miR-20b-5p	DIS3L	Cytokinesis	21572407
MIRT542144	hsa-miR-20b-5p	DIS3L	Cytokinesis	27292025
MIRT542145	hsa-miR-20a-5p	DIS3L	Cytokinesis	21572407
MIRT542145	hsa-miR-20a-5p	DIS3L	Cytokinesis	27292025
MIRT542146	hsa-miR-17-5p	DIS3L	Cytokinesis	21572407
MIRT542146	hsa-miR-17-5p	DIS3L	Cytokinesis	27292025
MIRT542147	hsa-miR-106b-5p	DIS3L	Cytokinesis	21572407
MIRT542147	hsa-miR-106b-5p	DIS3L	Cytokinesis	27292025
MIRT542148	hsa-miR-106a-5p	DIS3L	Cytokinesis	21572407
MIRT542148	hsa-miR-106a-5p	DIS3L	Cytokinesis	27292025
MIRT543882	hsa-miR-223-5p	AIMP1	Development Process	21572407
MIRT548130	hsa-miR-203a-3p	GAS1	Development Process	21572407
MIRT548132	hsa-miR-500a-5p	GAS1	Development Process	21572407
MIRT548417	hsa-miR-103a-3p	EML4	Cytokinesis	21572407
MIRT548417	hsa-miR-103a-3p	EML4	Cytokinesis	20371350
MIRT548874	hsa-miR-411-5p	CEP55	Development Process	21572407
MIRT548874	hsa-miR-411-5p	CEP55	Development Process	20371350
MIRT549232	hsa-miR-499a-5p	AXIN2	Embryo Development	21572407
MIRT549233	hsa-miR-208b-3p	AXIN2	Embryo Development	21572407
MIRT550319	hsa-miR-574-5p	SMAD9	Development Process	21572407
MIRT550324	hsa-miR-223-5p	SMAD9	Development Process	21572407
MIRT550430	hsa-miR-940	ORC4	Cytokinesis	21572407
MIRT551462	hsa-miR-23a-3p	TRIM59	Cytokinesis	21572407

MIRT553232	hsa-miR-18a-5p	TWF1	Cytokinesis	21572407
MIRT553233	hsa-miR-18b-5p	TWF1	Cytokinesis	21572407
MIRT553953	hsa-miR-197-3p	SS18	Development Process	21572407
MIRT555170	hsa-miR-222-3p	PTBP3	Development Process	21572407
MIRT555561	hsa-miR-101-3p	PLEKHA1	Reproductive Process	21572407
MIRT558366	hsa-miR-198	DIDO1	Cytokinesis	21572407
MIRT558366	hsa-miR-198	DIDO1	Cytokinesis	20371350
MIRT558368	hsa-miR-144-3p	DIDO1	Cytokinesis	21572407
MIRT558368	hsa-miR-144-3p	DIDO1	Cytokinesis	20371350
MIRT558369	hsa-miR-101-3p	DIDO1	Cytokinesis	21572407
MIRT558369	hsa-miR-101-3p	DIDO1	Cytokinesis	20371350
MIRT558373	hsa-miR-409-3p	DIDO1	Cytokinesis	21572407
MIRT558373	hsa-miR-409-3p	DIDO1	Cytokinesis	20371350
MIRT558788	hsa-miR-199a-3p	CEP55	Development Process	21572407
MIRT558927	hsa-miR-223-5p	CBX1	Cytokinesis	21572407
MIRT558935	hsa-miR-93-5p	CBX1	Cytokinesis	21572407
MIRT558938	hsa-miR-20b-5p	CBX1	Cytokinesis	21572407
MIRT558939	hsa-miR-20a-5p	CBX1	Cytokinesis	21572407
MIRT558940	hsa-miR-17-5p	CBX1	Cytokinesis	21572407
MIRT558941	hsa-miR-106b-5p	CBX1	Cytokinesis	21572407
MIRT558942	hsa-miR-106a-5p	CBX1	Cytokinesis	21572407
MIRT559273	hsa-miR-1248	B3GNT2	Development Process	21572407
MIRT559273	hsa-miR-1248	B3GNT2	Development Process	20371350
MIRT559277	hsa-miR-183-5p	B3GNT2	Development Process	21572407
MIRT559277	hsa-miR-183-5p	B3GNT2	Development Process	20371350
MIRT559796	hsa-miR-181b-5p	ZNF415	Cytokinesis	20371350
MIRT559797	hsa-miR-181a-5p	ZNF415	Cytokinesis	20371350
MIRT559930	hsa-miR-7-5p	SOD2	Development Process	20371350
MIRT561387	hsa-miR-103a-3p	TWF1	Cytokinesis	20371350
MIRT562711	hsa-miR-199a-5p	ZNF415	Cytokinesis	20371350
MIRT563320	hsa-miR-376c-3p	ORC4	Cytokinesis	20371350
MIRT567225	hsa-miR-215-5p	ID4	Reproductive Process	20371350
MIRT570516	hsa-miR-574-5p	SLC7A11	Development Process	20371350
MIRT570516	hsa-miR-574-5p	SLC7A11	Development Process	21572407
MIRT607626	hsa-miR-574-5p	TRIOBP	Embryo Development	24906430
MIRT607927	hsa-miR-122-5p	ANG	Reproductive Process	24906430
MIRT609030	hsa-miR-574-5p	EP300	Embryo Development	24906430
MIRT609030	hsa-miR-574-5p	EP300	Embryo Development	27418678
MIRT609369	hsa-miR-9-5p	SOD2	Development Process	23824327
MIRT612153	hsa-miR-125b-5p	SIX1	Embryo Development	23824327

MIRT612153	hsa-miR-125b-5p	SIX1	Embryo Development	19536157
MIRT612153	hsa-miR-125b-5p	SIX1	Embryo Development	27418678
MIRT612154	hsa-miR-125a-5p	SIX1	Embryo Development	23824327
MIRT612154	hsa-miR-125a-5p	SIX1	Embryo Development	19536157
MIRT612154	hsa-miR-125a-5p	SIX1	Embryo Development	27418678
MIRT612819	hsa-miR-601	KLHL3	Development Process	23824327
MIRT613136	hsa-miR-145-5p	DUSP6	Development Process	23824327
MIRT615941	hsa-miR-378a-5p	SORD	Reproductive Process	23824327
MIRT620859	hsa-miR-4772-3p	SERPING1	Development Process	23824327
MIRT623904	hsa-miR-1248	FOXK2	Development Process	23824327
MIRT624076	hsa-miR-129-5p	EBF1	Development Process	23824327
MIRT624076	hsa-miR-129-5p	EBF1	Development Process	27418678
MIRT625499	hsa-miR-940	SMAD9	Development Process	23824327
MIRT626269	hsa-miR-330-5p	EPHB3	Development Process	23824327
MIRT626269	hsa-miR-330-5p	EPHB3	Development Process	19536157
MIRT626270	hsa-miR-326	EPHB3	Development Process	23824327
MIRT626270	hsa-miR-326	EPHB3	Development Process	19536157
MIRT626362	hsa-miR-4772-3p	DIS3L	Cytokinesis	23824327
MIRT628683	hsa-miR-24-3p	TRAF3IP1	Embryo Development	23824327
MIRT630219	hsa-miR-24-3p	SORD	Reproductive Process	23824327
MIRT630232	hsa-miR-152-3p	SORD	Reproductive Process	23824327
MIRT630238	hsa-miR-216a-5p	SORD	Reproductive Process	23824327
MIRT640564	hsa-miR-182-5p	CPE	Development Process	23824327
MIRT640568	hsa-miR-24-3p	CPE	Development Process	23824327
MIRT640575	hsa-miR-1972	CPE	Development Process	23824327
MIRT640646	hsa-miR-429	IGF2	Embryo Development	23824327
MIRT640647	hsa-miR-200c-3p	IGF2	Embryo Development	23824327
MIRT640649	hsa-miR-200b-3p	IGF2	Embryo Development	23824327
MIRT641421	hsa-miR-128-3p	SERPING1	Development Process	23824327
MIRT642822	hsa-miR-23a-3p	SOD2	Development Process	23824327
MIRT643598	hsa-miR-150-5p	SPAG16	Reproductive Process	23824327
MIRT649030	hsa-miR-500a-5p	SLC1A2	Development Process	23824327
MIRT649055	hsa-miR-663a	SLC1A2	Development Process	23824327
MIRT653254	hsa-miR-1248	SORD	Reproductive Process	23824327
MIRT655608	hsa-miR-215-5p	ORC4	Cytokinesis	23824327
MIRT655609	hsa-miR-192-5p	ORC4	Cytokinesis	23824327
MIRT655612	hsa-miR-145-5p	ORC4	Cytokinesis	23824327
MIRT655612	hsa-miR-145-5p	ORC4	Cytokinesis	27292025
MIRT655813	hsa-miR-195-5p	NOTCH2	Development Process	22473208
MIRT655813	hsa-miR-195-5p	NOTCH2	Development Process	23824327

MIRT655814	hsa-miR-15b-5p	NOTCH2	Development Process	22473208
MIRT655814	hsa-miR-15b-5p	NOTCH2	Development Process	23824327
MIRT655815	hsa-miR-15a-5p	NOTCH2	Development Process	22473208
MIRT655815	hsa-miR-15a-5p	NOTCH2	Development Process	23824327
MIRT668298	hsa-miR-940	FOSL2	Development Process	23824327
MIRT668298	hsa-miR-940	FOSL2	Development Process	19536157
MIRT668302	hsa-miR-133a-3p	FOSL2	Development Process	23824327
MIRT668310	hsa-miR-133b	FOSL2	Development Process	23824327
MIRT670996	hsa-miR-24-3p	PTGIS	Reproductive Process	23824327
MIRT671040	hsa-miR-24-3p	SS18	Development Process	23824327
MIRT671574	hsa-miR-24-3p	FOSL2	Development Process	23824327
MIRT673600	hsa-miR-26a-5p	HPSE	Development Process	23824327
MIRT673600	hsa-miR-26a-5p	HPSE	Development Process	23313552
MIRT673609	hsa-miR-4772-3p	HPSE	Development Process	23824327
MIRT673629	hsa-miR-24-3p	PPM1D	Cytokinesis	23824327
MIRT674099	hsa-miR-143-3p	PLEKHA1	Reproductive Process	23824327
MIRT674571	hsa-miR-24-3p	KIF3A	Development Process	23824327
MIRT675483	hsa-miR-24-3p	SLC1A2	Development Process	23824327
MIRT675483	hsa-miR-24-3p	SLC1A2	Development Process	23313552
MIRT676625	hsa-miR-150-5p	CSNK1E	Development Process	23824327
MIRT680942	hsa-miR-940	EVC	Development Process	23706177
MIRT682667	hsa-miR-940	CASP8	Embryo Development	23706177
MIRT683772	hsa-miR-940	CPE	Development Process	23313552
MIRT685941	hsa-miR-150-5p	PTGIS	Reproductive Process	23313552
MIRT685960	hsa-miR-373-3p	PTGIS	Reproductive Process	23313552
MIRT685961	hsa-miR-372-3p	PTGIS	Reproductive Process	23313552
MIRT685963	hsa-miR-302d-3p	PTGIS	Reproductive Process	23313552
MIRT685967	hsa-miR-93-5p	PTGIS	Reproductive Process	23313552
MIRT685970	hsa-miR-20b-5p	PTGIS	Reproductive Process	23313552
MIRT685971	hsa-miR-20a-5p	PTGIS	Reproductive Process	23313552
MIRT685972	hsa-miR-17-5p	PTGIS	Reproductive Process	23313552
MIRT685973	hsa-miR-106b-5p	PTGIS	Reproductive Process	23313552
MIRT685974	hsa-miR-106a-5p	PTGIS	Reproductive Process	23313552
MIRT686480	hsa-miR-1254	TRIOBP	Embryo Development	23313552
MIRT686484	hsa-miR-150-5p	TRIOBP	Embryo Development	23313552
MIRT686496	hsa-miR-373-3p	TRIOBP	Embryo Development	23313552
MIRT686497	hsa-miR-372-3p	TRIOBP	Embryo Development	23313552
MIRT686499	hsa-miR-302d-3p	TRIOBP	Embryo Development	23313552
MIRT686503	hsa-miR-17-5p	TRIOBP	Embryo Development	23313552
MIRT686504	hsa-miR-20a-5p	TRIOBP	Embryo Development	23313552

MIRT686507	hsa-miR-106b-5p	TRIOBP	Embryo Development	23313552
MIRT686508	hsa-miR-20b-5p	TRIOBP	Embryo Development	23313552
MIRT686509	hsa-miR-106a-5p	TRIOBP	Embryo Development	23313552
MIRT686510	hsa-miR-93-5p	TRIOBP	Embryo Development	23313552
MIRT686821	hsa-miR-150-5p	SLC7A11	Development Process	23313552
MIRT686832	hsa-miR-373-3p	SLC7A11	Development Process	23313552
MIRT686833	hsa-miR-372-3p	SLC7A11	Development Process	23313552
MIRT686835	hsa-miR-302d-3p	SLC7A11	Development Process	23313552
MIRT686839	hsa-miR-93-5p	SLC7A11	Development Process	23313552
MIRT686842	hsa-miR-20b-5p	SLC7A11	Development Process	23313552
MIRT686843	hsa-miR-20a-5p	SLC7A11	Development Process	23313552
MIRT686844	hsa-miR-17-5p	SLC7A11	Development Process	23313552
MIRT686845	hsa-miR-106b-5p	SLC7A11	Development Process	23313552
MIRT686846	hsa-miR-106a-5p	SLC7A11	Development Process	23313552
MIRT690898	hsa-miR-203a-3p	TOP2A	Embryo Development	23313552
MIRT696580	hsa-miR-940	TTC21B	Development Process	23313552
MIRT698675	hsa-miR-181b-5p	TEF	Development Process	23313552
MIRT698676	hsa-miR-181a-5p	TEF	Development Process	23313552
MIRT702806	hsa-miR-183-5p	HPSE	Development Process	23313552
MIRT704098	hsa-miR-140-5p	DST	Cytokinesis	23313552
MIRT704532	hsa-miR-518a-5p	CNEP1R1	Cytokinesis	23313552
MIRT704541	hsa-miR-144-3p	CNEP1R1	Cytokinesis	23313552
MIRT704542	hsa-miR-101-3p	CNEP1R1	Cytokinesis	23313552
MIRT705555	hsa-miR-423-5p	ARID5B	Reproductive Process	23313552
MIRT707502	hsa-miR-10b-5p	AXL	Reproductive Process	21572407
MIRT707503	hsa-miR-10a-5p	AXL	Reproductive Process	21572407
MIRT715359	hsa-miR-181b-5p	VCAM1	Development Process	19536157
MIRT715363	hsa-miR-181a-5p	VCAM1	Development Process	19536157
MIRT716825	hsa-miR-142-3p	PSME4	Reproductive Process	19536157
MIRT717316	hsa-miR-22-3p	TRAF3IP1	Embryo Development	19536157
MIRT718465	hsa-miR-484	EED	Development Process	19536157
MIRT719013	hsa-miR-486-5p	HPGD	Reproductive Process	19536157
MIRT723985	hsa-miR-134-5p	SOX17	Embryo Development	19536157
MIRT724577	hsa-miR-1248	NOTCH2	Development Process	19536157
MIRT726208	hsa-miR-30e-5p	TWF1	Cytokinesis	22473208
MIRT726209	hsa-miR-30d-5p	TWF1	Cytokinesis	22473208
MIRT726210	hsa-miR-30b-5p	TWF1	Cytokinesis	22473208
MIRT726212	hsa-miR-20b-5p	TWF1	Cytokinesis	22473208
MIRT726213	hsa-miR-93-5p	TWF1	Cytokinesis	22473208
MIRT726214	hsa-miR-17-5p	TWF1	Cytokinesis	22473208

MIRT726782	hsa-miR-19a-3p	RASSF2	Development Process	22473208
MIRT727160	hsa-miR-181a-5p	MPP5	Development Process	22473208
MIRT727161	hsa-miR-181b-5p	MPP5	Development Process	22473208
MIRT727333	hsa-miR-19b-3p	KLHL3	Development Process	22473208
MIRT727334	hsa-miR-19a-3p	KLHL3	Development Process	22473208
MIRT727350	hsa-miR-19b-3p	KIF3A	Development Process	22473208
MIRT727351	hsa-miR-19a-3p	KIF3A	Development Process	22473208
MIRT727469	hsa-miR-27a-3p	IER3	Development Process	22473208
MIRT727471	hsa-miR-93-5p	IER3	Development Process	22473208
MIRT727472	hsa-miR-20b-5p	IER3	Development Process	22473208
MIRT727473	hsa-miR-17-5p	IER3	Development Process	22473208
MIRT727795	hsa-miR-30e-5p	EED	Development Process	22473208
MIRT727796	hsa-miR-30d-5p	EED	Development Process	22473208
MIRT727797	hsa-miR-30b-5p	EED	Development Process	22473208
MIRT727798	hsa-miR-30a-5p	EED	Development Process	22473208
MIRT727801	hsa-miR-181b-5p	EED	Development Process	22473208
MIRT727802	hsa-miR-181a-5p	EED	Development Process	22473208
MIRT727967	hsa-miR-93-5p	CNOT7	Cytokinesis	22473208
MIRT727969	hsa-miR-20b-5p	CNOT7	Cytokinesis	22473208
MIRT727970	hsa-miR-17-5p	CNOT7	Cytokinesis	22473208
MIRT731262	hsa-miR-129-5p	NR4A2	Development Process	26824182
MIRT731425	hsa-miR-130b-3p	DLL1	Embryo Development	28163094
MIRT731579	hsa-miR-376c-3p	NR5A2	Embryo Development	27049310
MIRT731625	hsa-miR-125a-5p	STAT3	Reproductive Process	26389681
MIRT731661	hsa-miR-223-3p	MYL9	Cytokinesis	27121304
MIRT732023	hsa-miR-146a-5p	NOTCH2	Development Process	27832663
MIRT732023	hsa-miR-146a-5p	NOTCH2	Development Process	28278511
MIRT732023	hsa-miR-146a-5p	NOTCH2	Development Process	26165719
MIRT732307	hsa-miR-181b-5p	SPP1	Reproductive Process	27192552
MIRT732491	hsa-miR-206	NR4A2	Development Process	25452104
MIRT732527	hsa-miR-483-5p	CKB	Development Process	25601461
MIRT732533	hsa-miR-34a-5p	ANK3	Development Process	25623948
MIRT732553	hsa-miR-223-3p	STAT3	Reproductive Process	26348153
MIRT732567	hsa-miR-103a-3p	SFRP4	Development Process	25820527
MIRT732605	hsa-let-7c-5p	STAT3	Reproductive Process	26851791
MIRT732901	hsa-miR-23a-3p	STAT3	Reproductive Process	26314966
MIRT732968	hsa-miR-206	TWF1	Cytokinesis	27435395
MIRT733075	hsa-miR-130a-3p	PPARGC1A	Development Process	25595716
MIRT733233	hsa-miR-338-5p	EFEMP1	Embryo Development	28292024
MIRT733293	hsa-miR-93-5p	ANG	Reproductive Process	28401709

MIRT733520	hsa-miR-128-3p	RICTOR	Embryo Development	27893811
MIRT733602	hsa-miR-106a-5p	MYB	Development Process	26265888
MIRT733738	hsa-miR-335-5p	DKK1	Embryo Development	26986081
MIRT733978	hsa-miR-34a-5p	CASP8	Embryo Development	28097098
MIRT733985	hsa-miR-34a-5p	GADD45A	Cytokinesis	28097098
MIRT734098	hsa-miR-101-3p	SRF	Embryo Development	28251884
MIRT734265	hsa-miR-9-5p	HES1	Embryo Development	27426040
MIRT734266	hsa-miR-9-5p	NOTCH2	Development Process	27426040
MIRT734349	hsa-miR-29b-3p	STAT3	Reproductive Process	26175849
MIRT734426	hsa-miR-191-5p	NOTCH2	Development Process	25992613
MIRT734721	hsa-let-7a-5p	STAT3	Reproductive Process	27932893
MIRT734758	hsa-let-7f-5p	POSTN	Development Process	25735962
MIRT734824	hsa-miR-152-3p	DKK1	Embryo Development	26400224
MIRT734902	hsa-miR-34a-5p	NR4A2	Development Process	27121375
MIRT734916	hsa-miR-10a-5p	BCL6	Reproductive Process	26590574
MIRT735023	hsa-miR-9-5p	DKK1	Embryo Development	27393149
MIRT735039	hsa-miR-34a-5p	DGKZ	Cytokinesis	28008152
MIRT735060	hsa-miR-31-5p	SLC1A2	Development Process	27995756
MIRT735061	hsa-miR-200c-3p	SLC1A2	Development Process	27995756
MIRT735175	hsa-miR-29a-5p	DKK1	Embryo Development	25846459
MIRT735309	hsa-miR-21-5p	CASP8	Embryo Development	26080425
MIRT736096	hsa-miR-1225-3p	GDF15	Development Process	26701625
MIRT736107	hsa-miR-122-5p	H1F0	Cytokinesis	26701625
MIRT736182	hsa-miR-1228-5p	CLU	Development Process	26701625
MIRT736558	hsa-miR-1254	CLU	Development Process	26701625
MIRT737007	hsa-miR-128-3p	GDF15	Development Process	26701625
MIRT737933	hsa-miR-15a-5p	CLU	Development Process	26701625
MIRT737955	hsa-miR-15b-5p	CLU	Development Process	26701625
MIRT737983	hsa-miR-16-5p	CLU	Development Process	26701625
MIRT738034	hsa-miR-181b-5p	H1F0	Cytokinesis	26701625
MIRT738622	hsa-miR-195-5p	CLU	Development Process	26701625
MIRT738675	hsa-miR-1972	LIF	Embryo Development	26701625
MIRT738675	hsa-miR-1972	LIF	Embryo Development	28735896
MIRT738995	hsa-miR-214-3p	KMT2B	Reproductive Process	26701625
MIRT739231	hsa-miR-24-3p	RRAS	Development Process	26701625
MIRT740494	hsa-miR-31-5p	KMT2B	Reproductive Process	26701625
MIRT741267	hsa-miR-326	CLU	Development Process	26701625
MIRT741325	hsa-miR-330-5p	CLU	Development Process	26701625
MIRT741433	hsa-miR-34a-5p	CRABP2	Embryo Development	26701625
MIRT743258	hsa-miR-423-5p	KMT2B	Reproductive Process	26701625

MIRT748686	hsa-miR-4772-3p	CXCL14	Development Process	26701625
MIRT748686	hsa-miR-4772-3p	CXCL14	Development Process	27418678
MIRT748686	hsa-miR-4772-3p	CXCL14	Development Process	28735896
MIRT760846	hsa-miR-940	SRF	Embryo Development	26701625
MIRT761361	hsa-miR-122-5p	VWC2	Development Process	27292025
MIRT761598	hsa-miR-1248	MFAP5	Development Process	27292025
MIRT762106	hsa-miR-127-3p	COTL1	Cytokinesis	27292025
MIRT762867	hsa-miR-150-5p	KIF3A	Development Process	27292025
MIRT764292	hsa-miR-26a-5p	EID2B	Development Process	27292025
MIRT764295	hsa-miR-26a-5p	KIF3A	Development Process	27292025
MIRT772886	hsa-miR-499a-5p	TRAF3IP1	Embryo Development	27292025
MIRT782903	hsa-miR-940	KIF3A	Development Process	27292025
MIRT782959	hsa-miR-940	VCAM1	Development Process	27292025
MIRT783223	hsa-miR-1254	KLHL3	Development Process	27418678
MIRT783462	hsa-miR-129-5p	CEP126	Cytokinesis	27418678
MIRT783480	hsa-miR-129-5p	EP300	Embryo Development	27418678
MIRT783605	hsa-miR-129-5p	STAR	Reproductive Process	27418678
MIRT783607	hsa-miR-129-5p	TAGLN	Development Process	27418678
MIRT783818	hsa-miR-143-3p	STAR	Reproductive Process	27418678
MIRT783999	hsa-miR-18a-5p	KLHL3	Development Process	27418678
MIRT784002	hsa-miR-18b-5p	KLHL3	Development Process	27418678
MIRT784361	hsa-miR-26a-5p	EP300	Embryo Development	27418678
MIRT785511	hsa-miR-411-5p	INTU	Embryo Development	27418678
MIRT787194	hsa-miR-518a-5p	CXCL14	Development Process	27418678
MIRT787194	hsa-miR-518a-5p	CXCL14	Development Process	28735896
MIRT790710	hsa-miR-518a-5p	CPE	Development Process	28735896
