# Supplementary material for: Development of a Novel Endometrial Signature Based on Endometrial microRNA for Determining the Optimal Timing for Embryo Transfer
Source: Biomedicines. 2024 Mar 21;12(3):700. doi: 10.3390/biomedicines12030700 (PMC10968378; doi:10.3390/biomedicines12030700)
Supplement: Supplementary file 1 [file biomedicines-12-00700-s001.zip › Table S3.pdf]

Supplemental Table S3. A list of differentially expressed miRNAs

	Fold Change (log2)	
**miRNA**	**120±5hrs vs 108±5hrs**	**120±5hrs vs 144±5hrs**
**hsa-miR-182-5p**		-0.6718
**hsa-miR-205-5p**		-1.0243
**hsa-miR-224-5p**	-1.0524	0.6347
**hsa-miR-17-3p**		0.6035
**hsa-miR-31-5p**		0.7222
**hsa-miR-382-5p**	0.759	0.834
**hsa-miR-593-5p**	0.9558	1.1594
**hsa-miR-596**		0.677
**hsa-miR-375-3p**		-1.1105
**hsa-miR-146a-5p**	-0.7946	
**hsa-miR-146b-5p**	-0.6263	
**hsa-miR-155-5p**	-0.8652	
**hsa-miR-20b-5p**	-0.853	
**hsa-miR-1973**	1.6413	
**hsa-miR-299-5p**	-0.694	
**hsa-miR-29a-5p**	-0.6691	
**hsa-miR-625-5p**	-0.6064	
**hsa-miR-95-3p**	-0.8184	
**hsa-miR-9-5p**	0.887	
**hsa-miR-31-3p**	0.8858	
**hsa-miR-1248**	1.2239	

The differentially expressed miRNAs were selected by the fold change >= ±1.5 with $p < 0.05$
