# Supplementary figures and images for: Development of a Novel Endometrial Signature Based on Endometrial microRNA for Determining the Optimal Timing for Embryo Transfer
Source: Biomedicines. 2024 Mar 21;12(3):700. doi: 10.3390/biomedicines12030700 (PMC10968378; doi:10.3390/biomedicines12030700)

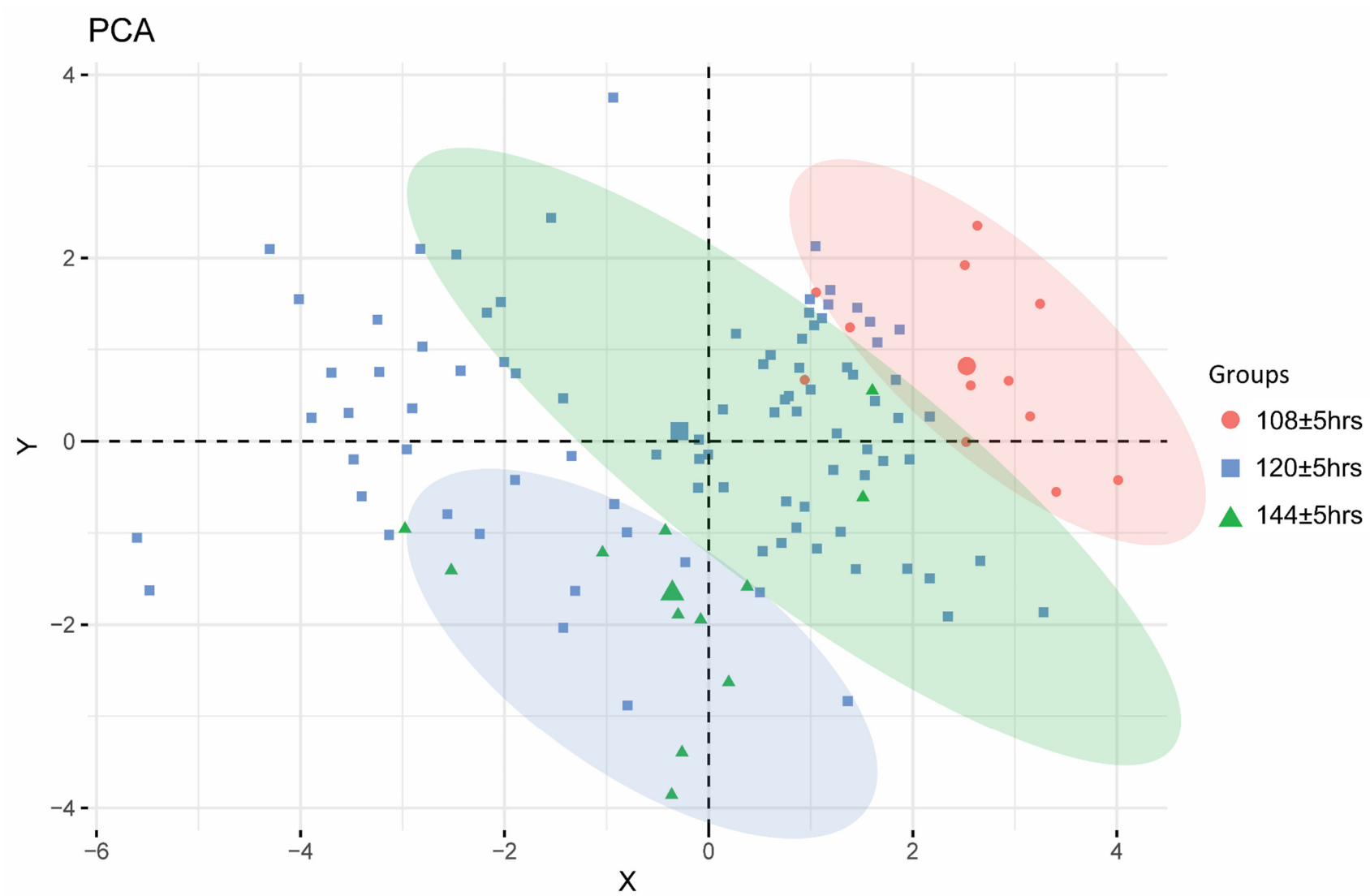

**Figure S3.** PCA-based cluster analysis based on the differentially expressed miRNA signatures.

Supplement: Supplementary file 1 [file biomedicines-12-00700-s001.zip › Figure S3.pdf]
